# Effects of dietary nitrate and folate supplementation on blood pressure in hypertensive Tanzanians: Design and baseline characteristics of a feasibility trial

**DOI:** 10.1016/j.conctc.2019.100472

**Published:** 2019-10-15

**Authors:** Navneet Kandhari, Meghna Prabhakar, Blandina T. Mmbaga, Jane Rogathi, Gloria Temu, William K. Gray, Stella-Maria Paddick, Richard Walker, Mario Siervo

**Affiliations:** a Faculty of Medical Sciences, Newcastle University, Claremont Road, Newcastle on Tyne, UK; b Kilimanjaro Christian Medical Centre and Kilimanjaro Christian Medical University College, Tanzania; c Kilimanjaro Clinical Research Institute, Moshi, Tanzania; d Northumbria Healthcare NHS Foundation Trust, North Shields, UK; e Institute of Health and Society, Newcastle University, Claremont Road, Newcastle on Tyne, UK; f Human Nutrition Research Centre, Institute of Cellular Medicine, Newcastle University, Newcastle on Tyne, NE2 4HH, UK; g School of Life Sciences, The University of Nottingham Medical School, Queen's Medical Centre, Nottingham, NG7 2UH, UK

**Keywords:** Dietary nitrate, Folate supplementation, Hypertension, Sub Saharan Africa

## Abstract

The burden of hypertension in Sub-Saharan African countries is rising. Low-cost and effective interventions are needed to mitigate these alarming trends. No evidence is available on the use of dietary nitrate for treating hypertension in African populations. The objectives of this study are to assess the feasibility and efficacy of using beetroot and folate as a combined dietary intervention to treat Tanzanian adults with pre- and mild to moderate hypertension. This was a three-arm double-blind, placebo-controlled, parallel randomised clinical trial conducted within the Hai Demographic Surveillance Site in the Kilimanjaro region in Tanzania. 48 participants were randomised to one of three groups for a 60-day intervention period. Group 1: Combined dietary intervention (beetroot juice and folate), Group 2: Single dietary intervention (beetroot juice and placebo capsule), and Group 3: Control group (placebo beetroot juice and placebo capsule). The primary outcome of the trial was to evaluate the feasibility of the study in a low-income setting. Trial assessments included resting clinic and ambulatory 24-hr blood pressure measurements, lifestyle and dietary questionnaires and collection of biological samples. Our cohort included 8 (16.7%) males and 40 (83.3%) females with mean age 60.7 years (SD 6.5). The mean (SD) BMI, clinic systolic blood pressure and ambulatory systolic blood pressure at baseline were 27.6 kg/m^2^ (5.4), 151.0 (19.4), and 140.4 (15.0) mmHg, respectively. Eight (16.7%) participants were classified as pre-hypertensive, 20 (41.7%) as stage-1 hypertensive, and 20 (41.7%) as stage-2 hypertensive. Overall, the results support the feasibility of a study of this nature within a hypertensive African population.

**Trial registration number:**

ISRCTN67978523.

## 1 Introduction

Hypertension is associated with a significant risk of cardiovascular morbidity and mortality worldwide [1]. The United Republic of Tanzania is a developing country within the East African region of Sub-Saharan Africa. Research indicates that current Tanzanian management strategies are struggling to control the burgeoning hypertension epidemic [2]. A recent study conducted by our group established a ‘*rule of sixths*’ in a cohort of hypertensive patients, where 2/6th of the cohort were previously diagnosed, 1/6th of those with a diagnosis were on treatment, and only 1/6th of those on treatment were adequately controlled [3].

Optimal control of hypertension remains challenging and the rising prevalence of hypertension in Tanzania calls for effective interventions. The need to develop a low-cost intervention that is both acceptable to local communities and effective either alone or alongside current medication is urgently warranted. Dietary interventions may be a valuable potential solution. The Dietary Approach to Stop Hypertension (DASH) diet is one of the best proven non-pharmacologic interventions for the prevention of hypertension [4] and the high inorganic nitrate content in the DASH diet may be responsible for this effect [5]. Recent studies have shown that dietary nitrate supplementation can reduce blood pressure and improve endothelial dysfunction [[6], [7], [8]]. Proposed mechanisms involve the generation of nitrite and subsequently nitric oxide, which promotes vasodilation to lower blood pressure [9].

Beetroot is a vegetable that is easily grown and naturally rich in dietary nitrate [10]. Studies utilising beetroot juice as a nitrate supplement have shown promising effects on reducing blood pressure [11]. One trial in healthy participants showed a reduced systolic and diastolic pressure of 10.4  mmHg and 8.0 mmHg respectively following a single dose after 3 h [9]. Similar benefits were seen in a sample of African-American women [12]. Additionally, folic acid is associated with mechanisms controlling the regulation of vascular tone (i.e., Nitric Oxide Synthase coupling, reduction of homocysteine) and its supplementation has been associated with improvements in endothelial function and blood pressure [[13], [14], [15]].

Poor adherence to medical treatment and lack of health infrastructures are key factors contributing to the poor control of hypertension in Tanzania [[16], [17], [18], [19]]. Dietary supplementation could potentially address these issues. Firstly, individuals may be more willing to take dietary supplements over anti-hypertensives due to the reduced side-effect profile and cultural perceptions of Western medicine, which could improve adherence to treatment. Secondly, using dietary supplements as an additional source of anti-hypertensive therapy may help alleviate the burden on already resource-limited healthcare systems, where a consistent supply of medication can often be unfeasible and expensive compared to obtaining dietary nitrate from foods that can be grown locally and sustainably. As beetroot and folate induce beneficial effects on BP via different mechanisms, a combined intervention may yield greater benefits compared to the consumption of each individually. To our knowledge, a combined dietary intervention focussed on folic acid and inorganic nitrate has not been conducted, especially in an African setting. Therefore, the aim of our study was to determine the feasibility of using beetroot and folate to lower blood pressure, and its efficacy for use in this setting.

## 2 Methods

### 2.1 Study design

Our study was a three-arm, placebo-controlled, double blind, randomised clinical trial (RCT) that was conducted in the Hai Demographic Surveillance Site (DSS) in the Kilimanjaro region of Northern Tanzania. Participants were assessed at Hai District Hospital and Kware village dispensary and samples were processed and stored at the Kilimanjaro Clinical Research Institute (KCRI). Recruitment started in March 2018, with trial assessments beginning in May and finishing in August.

In order to measure the effects of dietary intervention, we utilised clinic and ambulatory blood pressure measurements to examine changes in systolic and diastolic blood pressure. Blood and saliva samples were collected to evaluate compliance to the interventions (folic acid and nitrate) and measure biomarkers of nitric oxide production. The study comprised of four phases: (1) Recruitment, (2) Screening, (3) Randomisation and Baseline, and (4) Intervention. Eligible participants were randomised into one of three groups for an intervention period of 60 days:

• Group 1–Combined Intervention Group (High-Nitrate Beetroot and Folic Acid)
• Group 2–Single Intervention Group (High-Nitrate Beetroot and Placebo)
• Group 3–Control Group (Nitrate-Depleted Beetroot and Placebo)

The feasibility and acceptability of the intervention was evaluated through one-to-one qualitative interviews and participant compliance to the interventions and measurement protocols.

### 2.2 Study objectives

The primary objective of the study was to establish the feasibility, acceptability of intervention by participants and retention rate of the study. Secondary objectives included changes in 24-hr ambulatory blood pressure, whole-body nitric oxide production, homocysteine, C Reactive Protein, folate and plasma nitrate and salivary nitrate and nitrite concentrations.

### 2.3 Sample size

A formal statistical calculation of the sample size was not possible due to the lack of data on the effects of dietary nitrate on cardio-metabolic outcomes in sub-Saharan African populations. Therefore, we calculated our sample size using the approach for pilot trials proposed by Whitehead et al. [20]. In addition, we also used data from a published trial testing the effects of dietary nitrate on blood pressure in hypertensive British patients to further support the appropriateness of the sample size calculation [21]. The trial reported a significant effect of inorganic nitrate supplementation on systolic 24hr ABPM after 6 weeks of supplementation in 68 drug naïve hypertensive patients. The difference in systolic BP between the nitrate and placebo group was −7.7  mmHg (95%CI 4.1–11.2) with an effect size of 0.39. Applying a two-sided ANOVA model for repeated measures, we estimated that 14 participants per group (total sample size: 42) would be sufficient to detect a significant difference between placebo and nitrate intervention group with an alpha level of 0.05 and 80% power. A sample size of 15 participants per arm would allow the detection of a medium effect size (δ = 0.30–0.70) with 80% power and an alpha level less than 0.05. Therefore our study aimed to recruit 48 patients (16 per group) to allow for 10% drop out rate during the study.

### 2.4 Eligibility criteria

Participants were eligible if they were aged between 50 and 70 years, non-smokers and had an average systolic blood pressure between 130 and 170  mmHg and a BMI ranging from 18.0 to 40.0 kg/m^2^. Exclusion criteria were determined by factors likely to affect the study outcomes due to their influence on cardio-metabolic function, vascular function, and participant compliance. A list of all the inclusion and exclusion criteria can be seen in Appendix 1.

### 2.5 Recruitment

Census enumerators, individuals with a formal role and experience in conducting census and research work in their village of residence, were trained in accordance with the study protocol and identified local participants by convenience sampling. They obtained basic contact details, and a resting blood pressure measurement and invited willing participants to a full screening assessment at either of the two research sites: the Hai District Hospital or Kware village dispensary.

### 2.6 Screening

Two assessments were conducted during the screening phase (A and B). At screening A, eligibility was assessed according to the study eligibility criteria (see above). This assessment included anthropometric measurements, clinic blood pressure measurements (CBPMs), electrocardiography, and a brief medical history concerning drugs, diet and smoking. If eligible and willing to participate in the study, participants were invited to screening B where pre-baseline and feasibility data regarding lifestyle habits, knowledge of hypertension, and attitudes to the intervention were evaluated.

### 2.7 Randomisation and Baseline

Forty-eight eligible hypertensive individuals were recruited and consented to participate and randomised as they were enrolled in the trial. Participants were anonymised using three letter codes. Block-randomisation (4 participants per block) was used to generate a random sequence of codes for each intervention (Group 1, 2 or 3) and participants were assigned into one of the three arms of the trial according to their group number. Participants and the research team were blinded to the intervention. The head of the laboratory at Kilimanjaro Clinical Research Institute (KCRI) was responsible for the list of unblinded participants and dispensing of the interventions to ensure blinding of the trial was maintained. After randomisation, baseline visits were conducted and the interventions were dispensed.

### 2.8 Intervention

Following randomisation and baseline measures, 16 participants were assigned to each one of the three intervention groups for a period of 60 days. Measurements were taken at the research sites at baseline (day 0), interim (day 30), and at the end of the study (day 60). A telephone interview was conducted around 15 days after commencing the intervention to assess participant adherence and any concerns. Participants were asked to maintain their usual dietary habits, physical activity level and alcohol and caffeinated drinks consumption during the trial. All capsules were stored in identical white containers showing the individual participant code, the number of capsules, expiry date, storage and prescription instructions. Subjects were provided with a form to record the time of consumption and any problems they had with the interventions.

*Group 1 – “Combined Intervention: Nitrate-rich beetroot juice and folate capsule”*.

Participants were given one bottle (70 ml) of concentrated beetroot juice (Beet It shots®, James White LTD, UK) and one capsule of folate to be taken every morning. The juice corresponded to an average supplementation of approximately 400 mg of inorganic nitrate per day, and each capsule contained 5 mg of folate (Folic Acid, 5 mg, Bio-Tech Pharmacal. Inc, USA).

*Group 2 – “Single Intervention: Nitrate rich beetroot juice and placebo capsule”*.

Participants were given the same bottle (70 ml) of concentrated beetroot juice as group 1 (Beet It shots®, James White LTD, UK), and one placebo capsule containing sucrose powder to be taken every morning.

*Group 3 – “Control Group: Placebo juice and placebo capsule”*.

Participants were given one bottle (70 ml) of nitrate-depleted beetroot juice (<1 mg of inorganic nitrate, James White LTD, UK) and a placebo capsule containing sucrose powder to be taken every morning. This juice corresponded to an average supplementation of <1 mg of nitrate, and had the same appearance, colour and taste as the nitrate rich beetroot juice. The placebo capsules had the colour and appearance of the folic acid capsules.

### 2.9 Study measures

See Table 1 for a summary of the data collected at each visit.

**Table 1:** Summary of the data and biological samples collected throughout the study. ABPM = Ambulatory Blood Pressure Measurement, CBPM = Clinic Blood Pressure Measurement, ECG = Electrocardiography, FBC = Full Blood Count, ONT = Oral Nitrate Test, IPAQ = International Physical Activity Questionnaire, LH = Lithium Heparin.

Data Collected	Time Point						
	Enumerator Visits	Screening A	Screening B	Baseline (Day 0)	Telephone Call	Interim (Day 30)	Final Visit (Day 60)
Socio-demographic details (Name, age, gender)	Y	Y			Participants were called at around 15 days to see if they were experiencing any difficulties with the intervention		
Contact Details (Phone, Address)	Y	y					
Resting Blood Pressure	Y						
Eligibility (FBC, ECG, Medical History)		Y					
Informed Consent			Y				
IPAQ			Y				Y
Anthropometry		Y		Y		Y	Y
CBPM		Y		Y		Y	Y
ABPM				Y			Y
LH Blood sample				Y		Y	Y
ONT				Y			Y
Salivary Strips				Y		Y	Y
Feasibility Questionnaires			Y				
Feedback Questionnaires (FQ)						Short FQ	Full FQ

#### 2.9.1 Anthropometric measurements

Height, weight, mid-upper arm circumference (MUAC) and waist circumference were measured. Height was measured using a wall tape measure and weight was measured using calibrated weighing scales. Participants were fully clothed and barefoot for these measurements. BMI was calculated using these data. Mid-upper arm circumference was measured at the mid-point of the olecranon and acromion and waist circumference was measured at the level of the umbilicus.

#### 2.9.2 Clinic Blood Pressure Measurement (CBPM)

Resting CBPMs were carried out in accordance with the World Health Organisation STEPS protocol [22] using a calibrated automatic OMRON M3 BP device. Participants were asked to rest in a sitting position for at least 15 min. BP was measured using an appropriate sized cuff (regular or large) on the left upper arm supported at the level of the heart. BP was taken three times with a 1-min break in between each measurement. The average of the second and third values was recorded and used in the study.

#### 2.9.3 Ambulatory 24-h blood pressure monitoring (ABPM)

Participants were fitted with a 24-h ABPMs (Mobil‐O‐Graph [23], Stolberg, Germany) consisting of an inflatable cuff attached to small monitoring device. This device met the European Society of Hypertension and Association for Advancement of Medical Instrument criteria for BP measurements [24,25]. The cuff was secured around the individual's left upper arm according to mid-upper arm circumference (small, 20–24 cm; medium, 24–32 cm; large, 32–38 cm). A practice reading was taken in order to activate the monitor. Readings were then taken every 30 min in the day from 08:00 to 20:00 and every hour overnight from 20:00 to 08:00 the subsequent day. The following day, the participant would return the monitor and the BP data were uploaded onto the computer software.

#### 2.9.4 International physical activity questionnaire (IPAQ)

The short English IPAQ [26] was used to assess daily activity levels of participants.

#### 2.9.5 Oral nitrate test (ONT)

Eight saliva samples were taken to measure whole-body NO production [27]. As part of the ONT, participants were given water bottles with a known nitrate concentration, and a low nitrate meal to eat between 13:00–13:30. Participants were then instructed to drink an oral labelled sodium nitrate dose of 4 mg of Na^15^NO_3_ (Sigma-Aldrich, UK) in 100 mL of distilled water that was provided. Two hours after this, participants could eat another low nitrate snack before collecting their saliva samples. The first samples were collected at the research sites under supervision, after which the participants were given the equipment to collect samples at home and return them the next day. In order to take the samples, participants were instructed to chew on a cotton ball and then place the ball into a syringe to extract the saliva into tubes. Samples were collected into 2 mL Eppendorf tube containing 3.7 μL of sodium hydroxide to prevent degradation of nitrate during the collection procedure. Samples were stored at −20 °C at KCRI before shipment to the UK once the trial was completed. Saliva samples were derivatized using the nitromesitylene method and the enrichment level of the tracer was determined using gas chromatography/mass spectrometry [28].

#### 2.9.6 Salivary strips

Berkeley® NO salivary strips [29] were used to measure salivary nitrite at the study visits to evaluate their validity as a measure of compliance to nitrate interventions. A strip was placed on the tongue which changes colour upon saliva exposure. The participant's salivary nitrite levels were graded against a colour wheel supplied by the manufacturer [Appendix 2].

#### 2.9.7 Blood samples

At screening A, all participants had their full blood count measured for haemoglobin level to screen for anaemia. For eligible participants, blood samples were collected in 9 ml lithium heparin tubes at each study visit. After collection, all lithium heparin blood samples were immediately labelled and kept in ice boxes before being transferred to KCRI, where they were centrifuged into three plasma aliquots and stored at −80 °C until shipment to the UK. These were analysed for nitrate, folate, C Reactive Protein, nitro-tyrosine and homocysteine levels.

#### 2.9.8 Feasibility questionnaire

At screening visit B, semi-structured interviews were conducted in Swahili and English. The survey was split into three major domains: (1) Hypertension knowledge and perceptions of own health, (2) Attitudes to medication & dietary intervention and (3) Acceptability of the study protocol. Open and closed questions were utilised throughout. The data collected from open questions were later analysed for reoccurring themes and categorised. Closed questions gave simple categorical and numeric data, and a Likert scale was used in some instances. Exploratory probes were used to ensure participants had answered fully. Clarifying probes were used to address any ambiguities created as a result of mistranslation from Swahili into English. The survey also collected demographic information, family history and details of diet. The feedback questionnaires used in the study are showed in the online supplementary material.

#### 2.9.9 Telephone call (Day 15)

Approximately 15 days into the intervention, participants were called to assess their compliance and report difficulties with the intervention using a standardised questionnaire.

#### 2.9.10 Feedback questionnaires

A short feedback questionnaire at the interim visit (day 30), and a longer questionnaire at the end of the study (day 60) were completed to evaluate the feasibility and acceptability of the intervention.

All questionnaires used in the study are showed in the supplementary material.

### 2.10 Ethics

Ethical approval was obtained from Newcastle University, Kilimanjaro Christian Medical Centre (KCMC) research ethics committee (REC) and the National Institute of Medical Research Ethics Committees in Tanzania. Verbal consent was obtained in the villages by the enumerators during the recruitment phase. After screening A, all participants were given their test results and had the results explained to them. At screening B visits, information packs were given and informed consent was obtained from eligible participants before enrolment into the study. For individuals who were unable to write their own signature, thumbprints were taken to acknowledge their consent to participate. Participants were informed that they had the right to refuse consent and withdraw from the study at any time without having to give a reason. Those who were excluded were given appropriate medical advice and then referred to local health services for follow up. Participants were reimbursed for their travel costs when attending the research sites.

### 2.11 Trial registration and SPIRIT guidelines

The trial has been registered on the ISRCTN registry (Trial Number: ISRCTN67978523). The manuscript has been written in adherence with the SPIRIT Guidelines for describing protocol of clinical trials [30].

### 2.12 Statistical analysis

Continuous variables were described as mean ± SD. Categorical variables were summarised using absolute number and percentage of cases. Chi-square test was used to evaluate differences between groups for categorical variables. One-way analysis of variance was used to evaluate differences between groups for continuous variables. A p value less than 0.05 was considered as statistically significant. Statistical analyses were carried out using SPSS software (Version 24, NY, USA).

## 3 Results

### 3.1 Recruitment flowchart

**Fig. 1:**
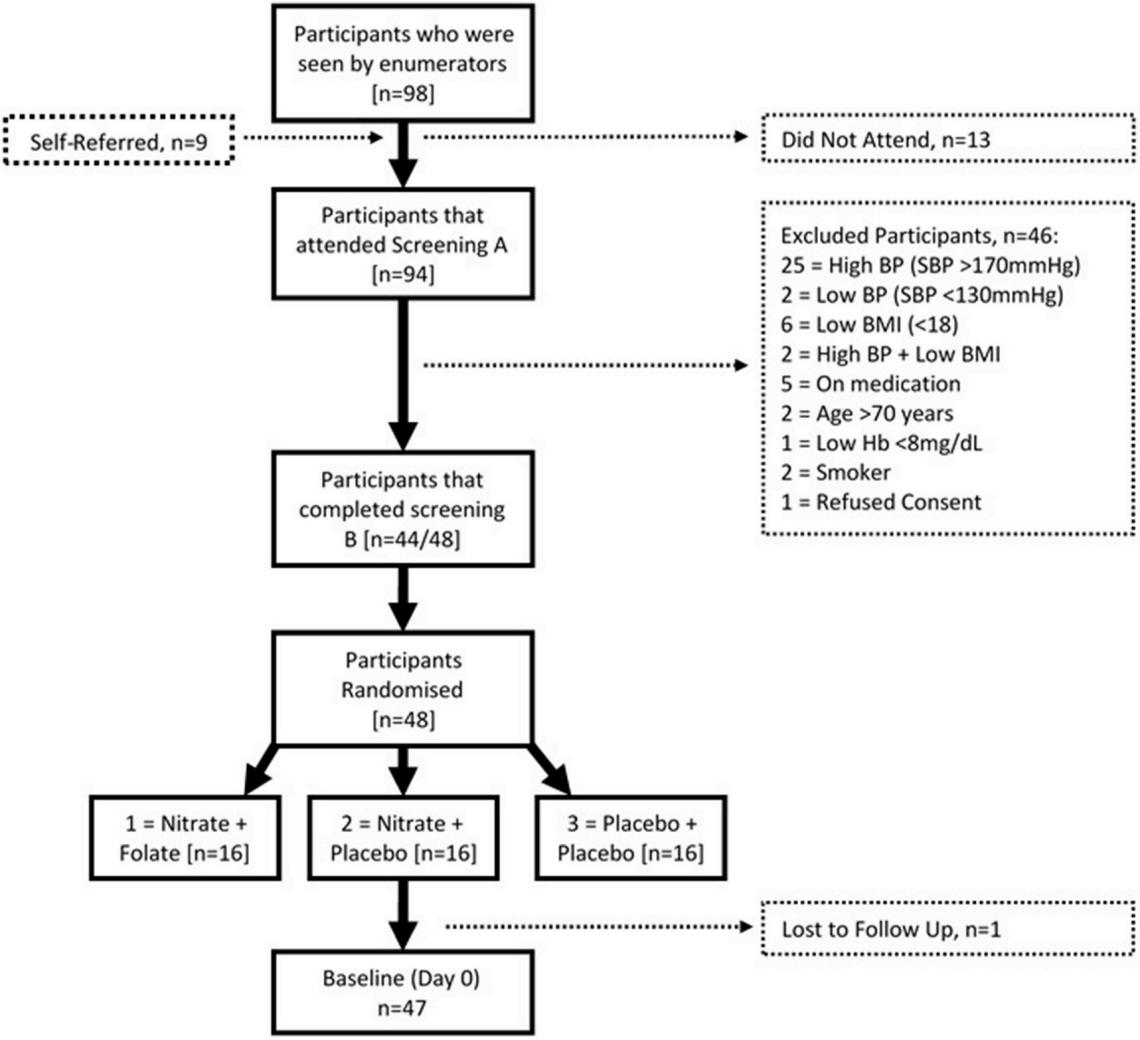
Flowchart of the study up to the baseline visit.

In total, 94 participants were screened over an 8-week period between March and April 2018, with baseline visits starting in May 2018. Forty-eight participants met the inclusion criteria and were successfully randomised to one of the three interventions. Four individuals were unable to complete the pre-baseline assessments at screening B. One participant was lost to follow up as they moved to a new city and so interventions were dispensed to 47 participants at baseline (Fig. 1).

### 3.2 Demographics

Baseline characteristics of the cohort are shown in Table 2. There were 8 (16.7%) males and 40 (83.3%) females with a mean age of 61.8 and 60.6 respectively. Forty (83.3%) belonged to the Chagga tribe and 28 (58.3%) members of the cohort had an occupation that involved farming. Active smokers were excluded. Apart from gender, groups were generally well matched across variables.

**Table 2:** Demographics of study cohort and groups of the trial. *1 = Chef, 1 = Car mechanic.

		Total (N = 48)	Group 1 (N = 16)	Group 2 (N = 16)	Group 3 (N = 16)	P
Age Years Median (Min-Max)		60 (50–70)	59 (50–70)	61 (50–70)	62 (50–70)	0.85
Gender (%)	Male	8 (16.7)	1 (6.3)	2 (12.5)	5 (31.3)	0.14
	Female	40 (83.3)	15 (93.8)	14 (87.5)	11 (68.8)	
Religion (%)	Islam	28 (58.3)	11 (68.8)	10 (62.5)	7 (43.8)	0.56
	Christian	17 (35.4)	4 (25.0)	6 (37.5)	7 (43.8)	
	No data	3 (6.3)	1 (6.3)	0 (0.0)	2 (12.5)	
Tribe (%)	Chagga	40 (83.3)	12 (75.0)	15 (93.8)	13 (81.3)	0.41
	Maasai	1 (2.1)	0 (0.0)	1 (6.3)	0 (0.0)	
	Haya	2 (4.2)	1 (6.3)	0 (0.0)	1 (6.3)	
	Nyaturu	1 (2.1)	1 (6.3)	0 (0.0)	0 (0.0)	
	Pare	4 (8.3)	2 (12.5)	0 (0.0)	2 (12.5)	
Marital status (%)	Married	27 (56.3)	8 (50.0)	10 (62.5)	9 (56.3)	0.12
	Widowed	13 (27.1)	6 (37.5)	2 (12.5)	5 (31.3)	
	Divorced	5 (10.4)	1 (6.3)	4 (25.0)	0 (0.0)	
	No data	3 (6.3)	1 (6.3)	0 (0.0)	2 (12.5)	
Village (%)	Boma Ng'ombe	4 (8.3)	1 (6.3)	1 (6.3)	2 (12.5)	0.84
	Gezaulole	1 (2.1)	1 (6.3)	0 (0.0)	0 (0.0)	
	Kengele	2 (4.2)	1 (6.3)	0 (0.0)	1 (6.3)	
	Kengereka	1 (2.1)	0 (0.0)	1 (6.3)	0 (0.0)	
	Kibaoni	2 (4.2)	1 (6.3)	0 (0.0)	1 (6.3)	
	Kware	29 (60.4)	10 (62.5)	10 (62.5)	9 (56.3)	
	Mkombozi	8 (16.7)	2 (12.5)	3 (18.8)	3 (18.8)	
	Udoro	1 (2.1)	0 (0.0)	1 (6.3)	0 (0.0)	
Occupation (%)	Farming/Animal Keeping	22 (45.8)	6 (37.5)	9 (56.3)	7 (43.8)	0.24
	Home-keeping	10 (20.8)	4 (25.0)	2 (12.5)	4 (25)	
	Farming and Trading	6 (12.5)	2 (12.5)	1 (6.3)	3 (18.8)	
	Trading	4 (8.3)	1 (6.3)	3 (18.8)	0 (0.0)	
	Unemployed	2 (4.2)	1 (6.3)	1 (6.3)	0 (0.0)	
	Skilled profession*	2 (4.2)	1 (6.3)	0 (0.0)	1 (6.3)	
	Missing Data	2 (4.2)	1 (6.3)	0 (0.0)	1 (6.3)	
Alcohol (%)	Never	19 (39.6)	6 (37.5)	6 (37.5)	7 (43.8)	0.83
	Ex-drinker	12 (25.0)	3 (18.8)	4 (25.0)	5 (31.3)	
	Currently Drinking	17 (35.4)	7 (43.8)	6 (37.5)	4 (25.0)	
Smoking (%)	Never	36 (75.0)	12 (75.0)	12 (75.0)	12 (75.0)	1.0
	Ex-smoker	12 (25.0)	4 (25.0)	4 (25.0)	4 (25.0)	

### 3.3 Baseline data

Hypertensive grading according to clinical guidelines can be found in Table 3. Table 4 shows the anthropometric and blood pressure characteristics of the cohort who started the intervention.

**Table 3:** Hypertensive Grading of Cohort according to NICE31 (National Institute for Health and Care Excellence) & ESC32 (European Society of Cardiology) guidelines.

	Baseline Hypertensive Grading			P
	Pre-hypertensive (%)	Grade 1 (%)	Grade 2 (%)	
Total (N = 48)	8 (16.7)	20 (41.7)	20 (41.7)	
Group 1 (N = 16)	1 (6.3)	6 (37.5)	9 (56.3)	0.14
Group 2 (N = 16)	5 (31.3)	6 (37.5)	5 (31.3)	
Group 3 (N = 16)	2 (12.5)	8 (50)	6 (37.5)

**Table 4:** Baseline data of cohort. BMI = body mass index, ABPM = Ambulatory Blood Pressure Measurement, CBPM = Clinic Blood Pressure Measurement, MAP = Mean Arterial Pressure. *ABPM n = 44 as 3 participants refused to perform the measurements.

Variable (Baseline)		Total (SD) n = 47	Group 1 (SD) n = 16	Group 2 (SD) n = 16	Group 3 (SD) n = 15	P
Anthropometry	Height (cm)	162.2 (8.3)	162.4 (7.4)	161.9 (7.9)	161.4 (9.3)	0.98
	Weight (kg)	72.3 (15.3)	76.6 (15.4)	69.0 (13.1)	71.4 (17.3)	0.36
	Upper Arm Circumference (cm)	31.2 (4.2)	32.6 (4.7)	30.3 (3.8)	30.7 (3.8)	0.24
	Waist Circumference (cm)	98.3 (11.0)	99.6 (10.0)	97.9 (11.5)	97.2 (12.2)	0.82
	BMI (kg/m^2^)	27.6 (5.4)	29.1 (5.8)	26.3 (4.7)	27.3 (5.7)	0.23
Blood Pressure (mmHg)	CBPM Systolic	151.0 (19.4)	155.8 (18.6)	150.0 (23.9)	147.0 (14.5)	0.45
	CBPM Diastolic	91.8 (11.7)	94.0 (10.5)	92.0 (15.0)	89.2 (9.0)	0.52
	CBPM Mean Arterial Pressure (MAP)	121.4 (14.1)	124.9 (13.0)	121.0 (18.7)	118.1 (8.6)	0.47
	*ABPM Systolic	140.4 (15.0)	141.9 (12.3)	131.6 (15.9)	137.0 (13.8)	0.55
	*ABPM Diastolic	88.4 (11.1)	90.2 (9.9)	81.2 (11.2)	82.7 (7.6)	0.08
	*ABPM MAP	112.3 (11.5)	113.9 (9.7)	104.3 (12.6)	107.6 (7.9)	0.27

**Fig. 2:**
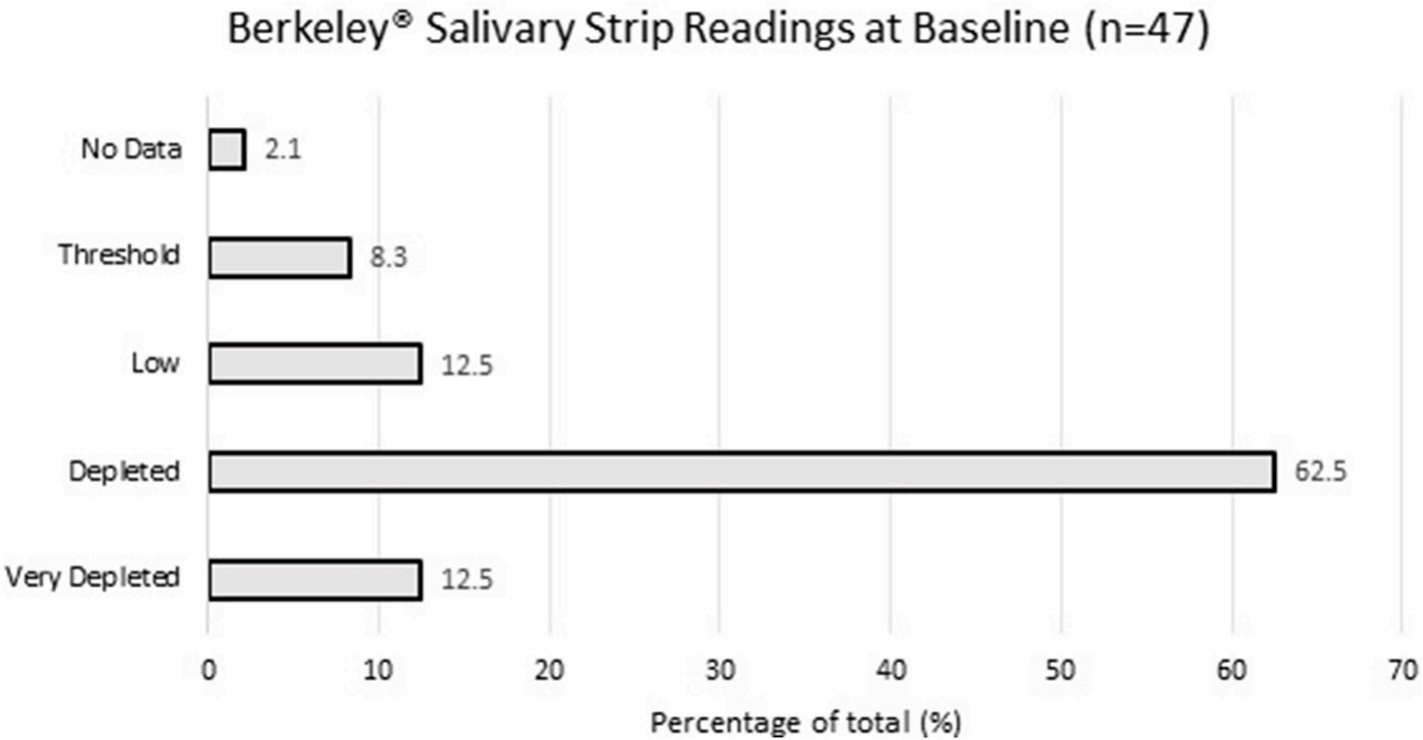
Salivary strip30 grading determined by Berkeley colour scoring on NO test strips: Very Depleted (<20), Depleted (20–109), Low (110–219), Threshold (220–434), Target (435–869), High (>870).

Forty-four participants completed the IPAQ. Most of the cohort had high MET (Metabolic Equivalents) scores, owing to high levels of weekly physical activity (Table 5). Twenty-four reported to not perform vigorous activity, resulting in a low LQ and median for vigorous activity scores. Thirty-nine (86.6%) were classified as having high levels of activity, with most time spent doing moderate activity such as farming or cultivating. Mean haemoglobin levels of the cohort were 14.1 ± 1.2  g/dL (range: 11.7–17.2  g/dL). Salivary strips were taken from 46 participants at baseline. No participants had a salivary nitrite grade of target or higher (Fig. 2).

**Table 5:** MET (Metabolic Equivalents) scores of study cohort (N = 44). Time spent when active is noted and multiplied by the energy demand of the corresponding activity (Walking = 3.3 METs, Moderate Activity = 4 METs, Vigorous Activity = 8 METs). Activity Classification: High = >3000 METs per week, Moderate = 600–3000 METs per week, Low = <600 METs per week.

	METs Score of Study Cohort (per week)			
	Walking	Moderate Activity	Vigorous Activity	Total METs
Lower Quartile (LQ)	396	1680	0	5752
Median	916	4920	0	10874
Upper Quartile (UQ)	2772	11760	6120	18925

## 4 Discussion

In this study, we have demonstrated the rationale, design and baseline data of the participants who took part in our dietary intervention trial. Important findings regarding our methodology and baseline data are discussed below.

Firstly, enumerators were an invaluable resource during the recruitment phase of our trial and provided valuable insight into cultural beliefs within the area. This helped adapt our study protocol to be more acceptable to the community. As an example, the initial role of the enumerators was to identify individuals who were likely to meet the study criteria and selectively invite them to screening in order to streamline the recruitment process. In practice however, this was not culturally acceptable. Individuals that the enumerators deemed unlikely to meet the study criteria would complain that it was unfair that other people were being selected over them when they were also suffering from various illnesses. Consequently, we decided to screen anyone seen by the enumerators even if they were already unsuitable for the study. Whilst this increased the overall time required for the screening phase, many individuals expressed their gratitude for our presence, and this could have actually helped bring more participants to the research sites. Indeed, nine individuals came to the Kware dispensary for screening after hearing about the study from others.

Our cohort was somewhat representative of the demographics of the Kilimanjaro region, in that most belonged to the Chagga tribe (83.3%) and were farmers (58.3%) [3,31]. However, females were overrepresented. Enumerators often reported that men were away working on farms whilst they were conducting visits. As a result, more women were seen at home and available for the study. Other studies have also reported that women tend to have better health seeking behaviours than men which may have contributed to this disparity [16]. To limit the effect this may have on results, this issue should be addressed in future studies. Monetary incentives could be utilised to act as a substitute for lost pay and conducting the study in the dry season would help, as many participants reported being more likely to be available as they would not be doing farm work then. Moreover, heavy rains during our study often limited road accessibility, and our research team noted that participants were significantly late when coming to clinic on rainy days. The dry season in the Kilimanjaro region is June-December [32,33]. Overall, the total number of individuals approached by enumerators or self-referred to the study was 107. Therefore, the ratio for participants approached to included was roughly 2:1. This ratio is important to consider if a larger sample size is needed for a future study.

The IPAQ was useful in assessing activity levels of the cohort. However, many participants found it difficult to quantify certain activities such as “*time spent sitting*” or they overestimated their times, as they explained they would farm and dig for 8 h without accounting for breaks. This may affect the quality of the data. Assessing activity levels was not a main aim for our study, but it is worth noting that many of these questions may need to be adapted should the IPAQ be utilised for a future study in this setting.

In comparison to the ONT, the salivary strips provided quick and easily interpretable results which could be immediately explained to participants and did not require laboratory storage. However, as they are graded from a colour scale, salivary strips may be subject to interpretive bias, and also cannot differentiate between participants of the same grade. To resolve this, a mobile application produced by the manufacturer can be used to interpret the strips and provide quantitative data. This could be employed in a future trial to increase the sensitivity of this test. Ultimately, if analysis by ozone-based chemiluminescence of the saliva samples collected at the same time gave similar results to the saliva strips, it would be more feasible to use the salivary strips in future studies, especially in this setting, for both cost and convenience.

### 4.1 Strength and limitations

To our knowledge, a RCT of this nature has never been conducted in this population and is completely unique for a Sub-Saharan African setting. Utilising the Hai DSS meant that we had a known high prevalence of hypertension in the area from previous research, and a well-defined population. These factors indicate that this population would be an ideal target for any future larger scale trials and thus increases the importance of our findings. Most importantly, this study has yielded novel findings of the acceptability of using assessment tools such as ABPMs, blood samples, ONT test, IPAQs and salivary strips within a Tanzanian population that was unfamiliar with them. We have also demonstrated that our study protocol was feasible in a rural cohort, which may be of benefit for other dietary intervention studies.

Certain factors may limit the generalizability of our results. Firstly, our study cohort was heavily gender biased towards women, and it is possible that men will have differing opinions on the acceptability of our study tools and intervention. Secondly, results may vary between villages that are within the same DSS. As our study participants were sampled from only two rural villages, the findings may not be wholly representative of other villages in the DSS, as well as other urbanized areas where the population would have differing lifestyle factors and dietary habits. Therefore, our results should be extrapolated to other SSA settings with careful consideration, as population lifestyles and diet may vary markedly. The timing of the interventions also coincided with Ramadan and over half of the participants were Muslim. It is important to recognize that effects of fasting may have had an influence on the BP measurements, although other studies investigating this influence have had mixed results [34].

### 4.2 Implications

Overall, this pilot study indicates that a nutritional intervention of this nature is possible within a hypertensive African population.

## Author contributions

MS is the guarantor of this work and, as such, had full access to all the data in the study and takes responsibility for the integrity of the data and the accuracy of the data analysis. MS and RW conceived and designed the study. NK, MP, JR acquired the data. NK, MP, RW and MS wrote the manuscript. All authors contributed to the analysis, discussion, and interpretation of data, and reviewed/critically edited the manuscript. All authors have read and approved the final manuscript.

## Funding

This project was funded by the UK Medical Research Council Confidence in Concept Funding scheme (Grant Number: BH171899).

## Declaration of competing interest

The authors have no conflicts of interest to declare.

## Data Availability

The dataset used to generate the baseline data has been attached to the manuscript.

## References

[1] Lozano R., Naghavi M., Foreman K. (2012). Global and regional mortality from 235 causes of death for 20 age groups in 1990 and 2010: a systematic analysis for the Global Burden of Disease Study 2010. The Lancet.

[2] Isangula Kahabi G., Meda John R. (2017). The burden of hypertension in the rural and urban populations of Tanzania: a review of trends, impacts and response. Tanzan. J. Health Sci..

[3] Dewhurst M.J., Dewhurst F., Gray W.K. (2012). The high prevalence of hypertension in rural-dwelling Tanzanian older adults and the disparity between detection, treatment and control: a rule of sixths?. J. Hum. Hypertens..

[4] Whelton P.K., Carey R.M., Aronow W.S. (2017). ACC/AHA/AAPA/ABC/ACPM/AGS/APhA/ASH/ASPC/NMA/PCNA guideline for the prevention, detection, evaluation, and management of high blood pressure in adults. A Report of the American College of Cardiology/American Heart Association Task Force on Clinical Practice Guidelines 2017.

[5] Hord N.G., Tang Y., Bryan N.S. (2009). Food sources of nitrates and nitrites: the physiologic context for potential health benefits. Am. J. Clin. Nutr..

[6] Ashor A.W., Lara J., Siervo M. (2017). Medium-term effects of dietary nitrate supplementation on systolic and diastolic blood pressure in adults: a systematic review and meta-analysis. J. Hypertens..

[7] Lara J., Ashor A.W., Oggioni C. (2016). Effects of inorganic nitrate and beetroot supplementation on endothelial function: a systematic review and meta-analysis. Eur. J. Nutr..

[8] Gee L.C., Ahluwalia A. (2016). Dietary nitrate lowers blood pressure: epidemiological, pre-clinical experimental and clinical trial evidence. Curr. Hypertens. Rep..

[9] Webb A.J., Patel N., Loukogeorgakis S. (2008). Acute blood pressure lowering, vasoprotective, and antiplatelet properties of dietary nitrate via bioconversion to nitrite. Hypertension.

[10] Pietro S. (2006). Nitrate in vegetables: toxicity, content, intake and EC regulation. J. Sci. Food Agric..

[11] Siervo M., Lara J., Ogbonmwan I. (2013). Inorganic nitrate and beetroot juice supplementation reduces blood pressure in adults: a systematic review and meta-analysis. J. Nutr..

[12] Bond V., Curry B.H., Adams R.G. (2014). Effects of nitrate supplementation on cardiovascular and autonomic reactivity in African-American females. ISRN Physiol..

[13] Wang W.-W., Wang X.-S., Zhang Z.-R. (2017). A meta-analysis of folic acid in combination with anti-hypertension drugs in patients with hypertension and hyperhomocysteinemia. Front. Pharmacol..

[14] Doshi S.N., McDowell I.F.W., Moat S.J. (2002). Folic acid improves endothelial function in coronary artery disease via mechanisms largely independent of homocysteine lowering. Circulation.

[15] Stanhewicz A.E., Kenney W.L. (2017). Role of folic acid in nitric oxide bioavailability and vascular endothelial function. Nutr. Rev..

[16] Kayima J., Wanyenze R.K., Katamba A. (2013). Hypertension awareness, treatment and control in Africa: a systematic review. BMC Cardiovasc. Disord..

[17] Mosha N.R., Mahande M., Juma A. (2017). Prevalence, awareness and factors associated with hypertension in North West Tanzania. Glob. Health Action.

[18] Mayige M., Kagaruki G., Ramaiya K. (2011). Non communicable diseases in Tanzania: a call for urgent action. Tanzan. J. Health Res..

[19] Maginga J., Guerrero M., Koh E. (2016). Hypertension control and its correlates among adults attending a hypertension clinic in Tanzania. J. Clin. Hypertens..

[20] Whitehead A.L., Julious S.A., Cooper C.L. (2016). Estimating the sample size for a pilot randomised trial to minimise the overall trial sample size for the external pilot and main trial for a continuous outcome variable. Stat. Methods Med. Res..

[21] Kapil V., Khambata R.S., Robertson A. (2015). Dietary nitrate provides sustained blood pressure lowering in hypertensive patients: a randomized, phase 2, double-blind, placebo-controlled study. Hypertension.

[22] WHO (2008). The WHO STEPS Instrument: the WHO STEPwise Approach to Chronic Disease Risk Factor Surveillance (STEPS).

[23] (2018). I.E.M. Mobil-O-Graph® PWA. https://www.iem.de/en/products/mobil-o-graph.html#pwa.

[24] Franssen P.M., Imholz B.P. (2010). Evaluation of the Mobil-O-Graph new generation ABPM device using the ESH criteria. Blood Press. Monit..

[25] Jones C.R., Yalor K., Chowienczyk P. (2000). A validation of the Mobil O Graph (version 12) ambulatory blood pressure monitor. Blood Press. Monit..

[26] IPAQ (2005). Guidelines for the Data Processing and Analysis of the "International Physical Activity Questionnaire.

[27] Siervo M., Jackson S.J., Bluck L.J.C. (2011). In-vivo nitric oxide synthesis is reduced in obese patients with metabolic syndrome: application of a novel stable isotopic method. J. Hypertens..

[28] Jackson S.J., Siervo M., Persson E. (2008). A novel derivative for the assessment of urinary and salivary nitrate using gas chromatography/mass spectrometry. Rapid Commun. Mass Spectrom..

[29] Life Berkeley (2018). Nitric oxide saliva test strips. https://www.berkeleylife.com/nitric-oxide-saliva-test-strips/.

[30] Chan A.-W., Tetzlaff J.M., Altman D.G. (2013). SPIRIT 2013 statement: defining standard protocol items for clinical trials. Ann. Intern. Med..

[31] National Bureau of Statistics (2016). Basic Demographic and Socio-Economic Profile - Kilimanjaro Region. 2012 Population and Housing Census.

[32] Bureau of Statistics Planning Commission, Macro International Inc (1997). Tanzania Demographic and Health Survey 1996 Calverton.

[33] Climate-DataOrg Climate: Kilimanjaro. https://en.climate-data.org/africa/tanzania/kilimanjaro-1714/.

[34] Trepanowski J.F., Bloomer R.J. (2010). The impact of religious fasting on human health. Nutr. J..

