# Supplementary material for: Effects of dietary nitrate and folate supplementation on blood pressure in hypertensive Tanzanians: Design and baseline characteristics of a feasibility trial
Source: Contemp Clin Trials Commun. 2019 Oct 15;16:100472. doi: 10.1016/j.conctc.2019.100472 (PMC6915790; doi:10.1016/j.conctc.2019.100472)

**APPENDICES & SUPPLEMENTARY MATERIAL**

[Appendix 1 - Exclusion Criteria 1](#_Toc4576573)

[Appendix 2 - Manufacturer Instructions & Colour Wheel for Berkeley Test® Strips 2](#_Toc4576574)

[Appendix 3 - Enumerator Questionnaire (English and Swahili) 4](#_Toc4576575)

[Appendix 4 – Screening Questionnaire 8](#_Toc4576576)

[Appendix 5 – Feasibility Questionnaire 10](#_Toc4576577)

[Appendix 6 - International Physical Activity Questionnaire (IPAQ) 15](#_Toc4576578)

[Appendix 7 – Telephone Questionnaire 17](#_Toc4576579)

[Appendix 8 – Short Feedback Questionnaire 19](#_Toc4576580)

[Appendix 9 – Baseline Visit Questionnaire 21](#_Toc4576581)

[Appendix 10 - 30-day Interim Visit 23](#_Toc4576582)

[Appendix 11 - Full Feedback Questionnaire 26](#_Toc4576583)

# Appendix 1 - Exclusion Criteria

- Smoking
- Ongoing participation in other clinical studies
- Physical disabilities that limit mobility
- Vegetarian diet (would have high nitrate intake)
- Inability to comply with study diet
- Significant weight change in the previous 3 months (> 5 kg)
- Active cancer or previous diagnosis of malignant cancer within the last 5 years
- Chronic or acute metabolic and inflammatory conditions (Rheumatoid Arthritis, inflammatory bowel diseases, severe liver disease)
- Type 1 or type 2 Diabetes treated with Insulin
- On medication (Diuretics, Oral corticosteroids, Laxatives, Anticoagulants, Nitrate derived agents, Anti-cholinergics and Anti-hypertensives such as Ace-inhibitors, Angiotensin receptor inhibitors, Beta blockers or Calcium channel antagonists)
- History of severe anaemia (Hb < 8mg/dL)
- Current diagnosis of severe infectious diseases (e.g HIV, malaria, hepatitis, yellow fever)
- History of major surgical operations.
- Participants taking hormonal therapies (oestrogens, thyroxine, progesterone, oral hypoglycaemic agents), antidyslipidaemic (statins) and psychiatric drugs (antidepressants, sedatives, antipsychotics) were excluded if they had either started or altered their dose within the last three months.

Appendix 2 - Manufacturer Instructions & Colour Wheel for Berkeley Test® Strips
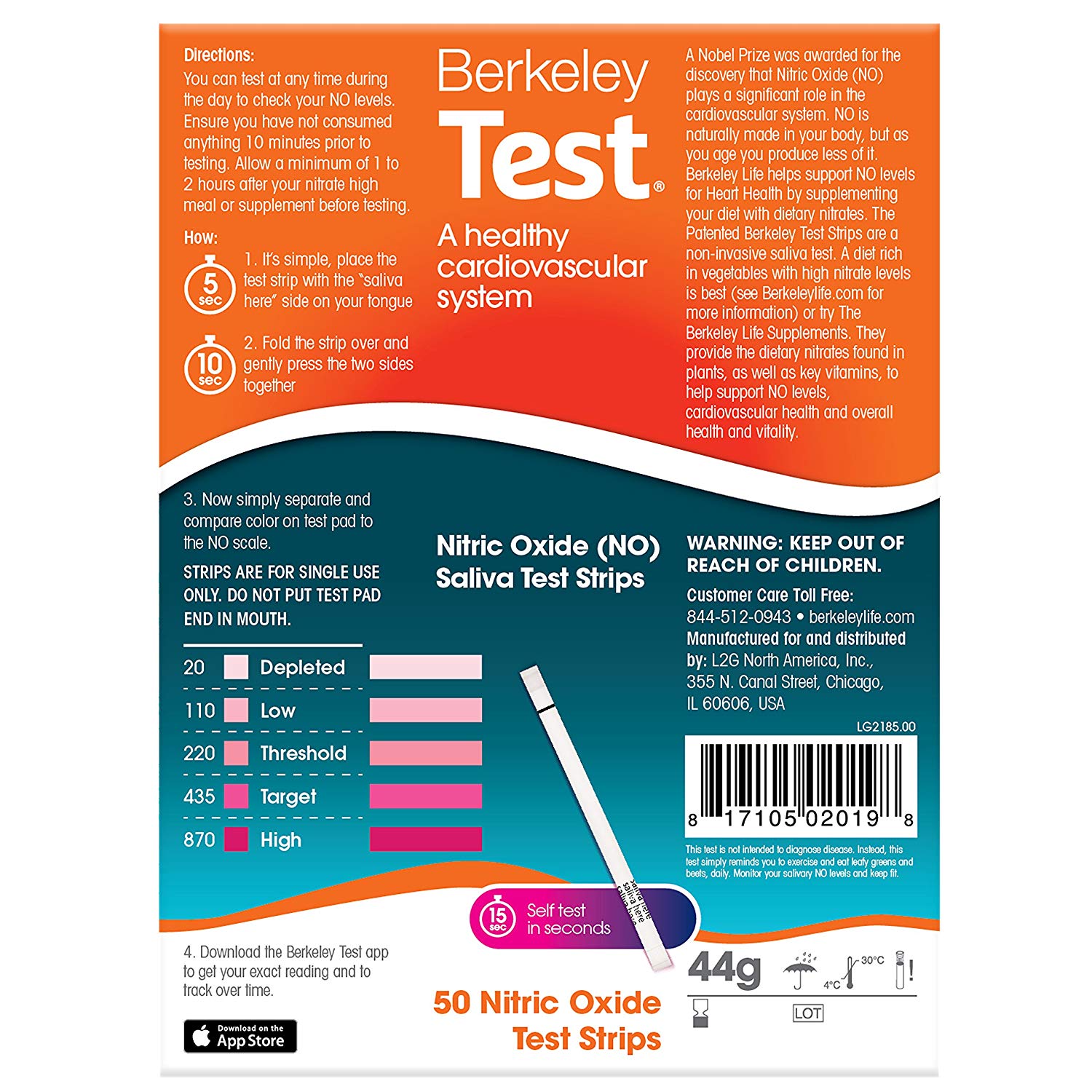


# Appendix 3 - Enumerator Questionnaire (English and Swahili)


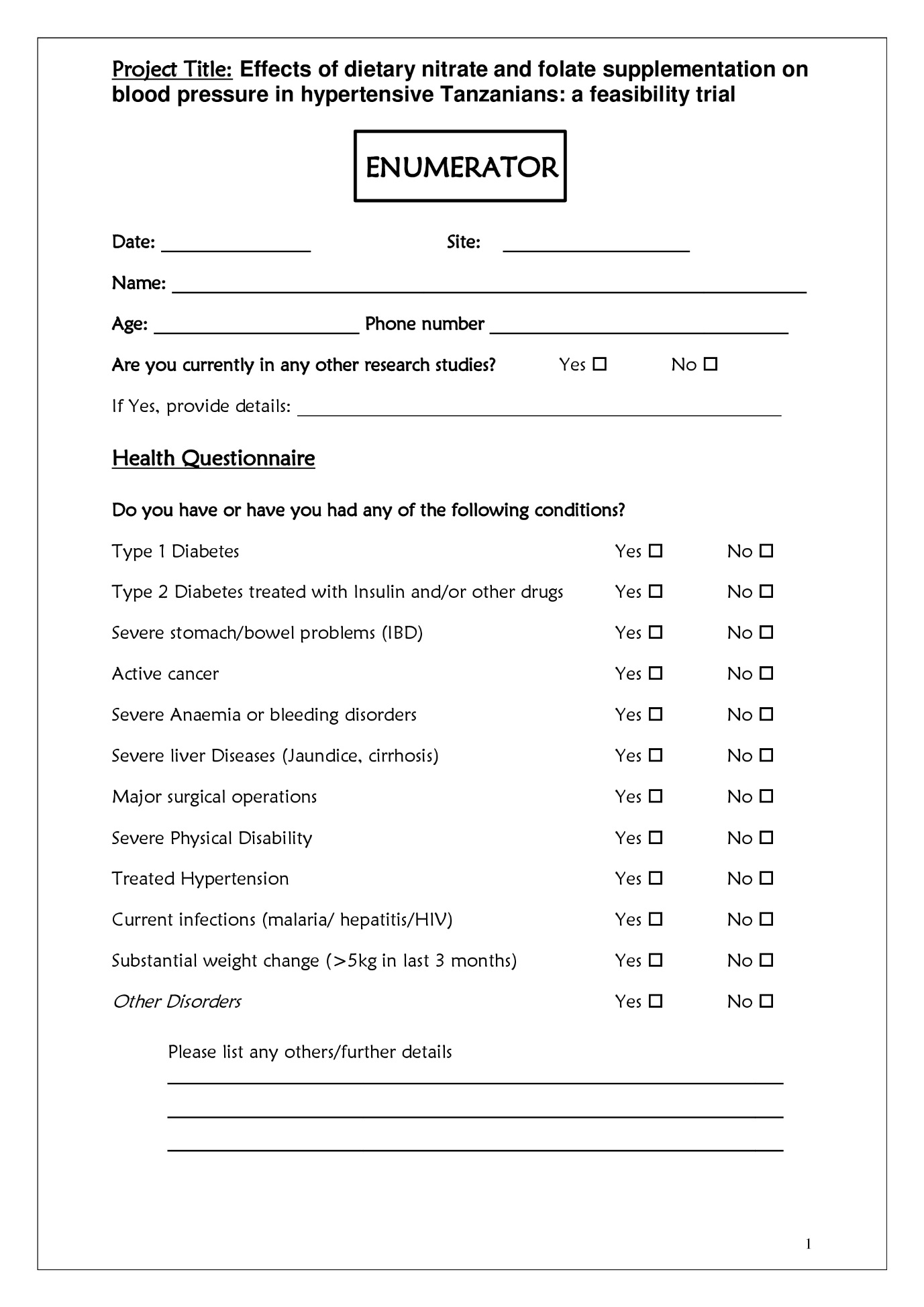


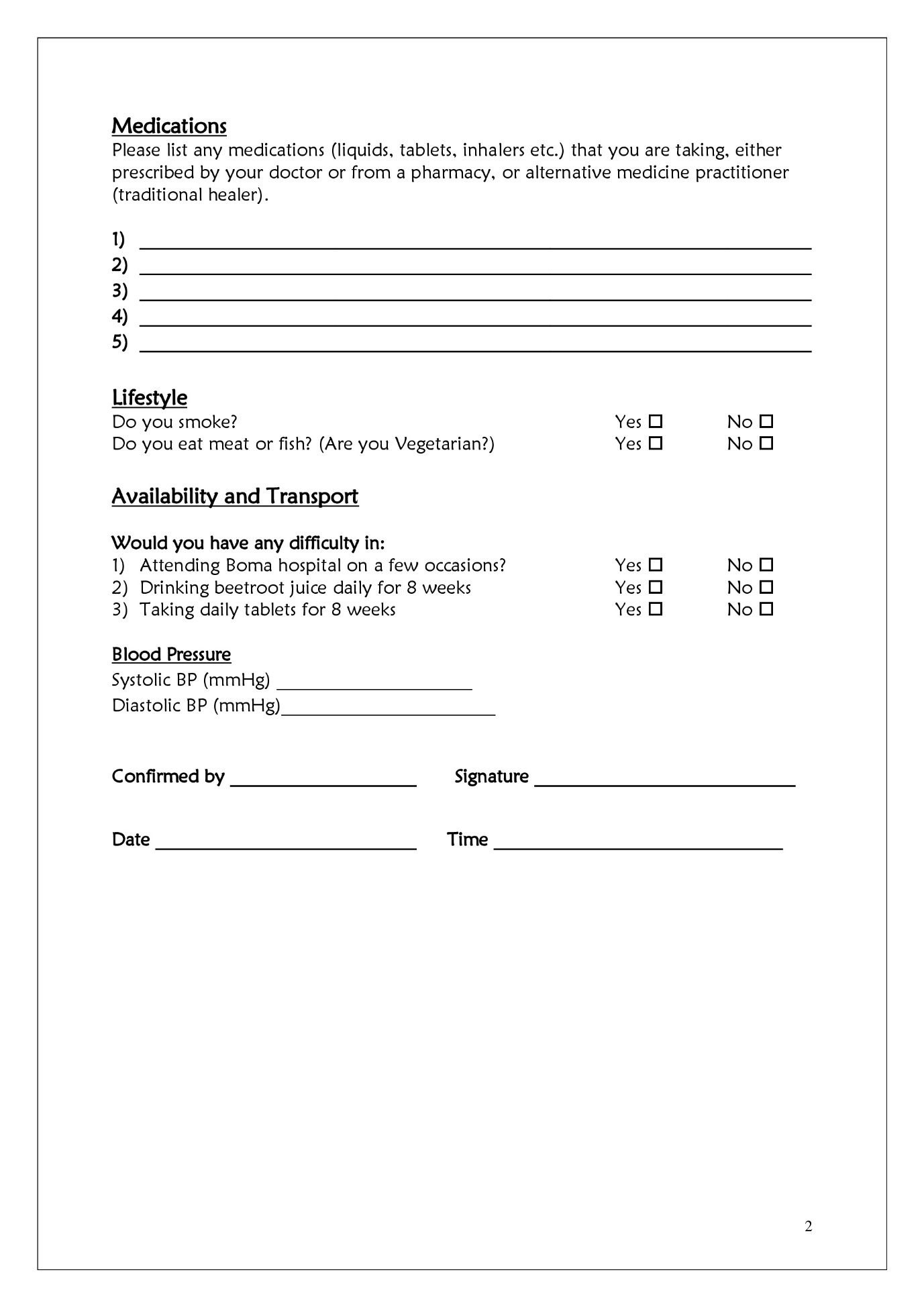


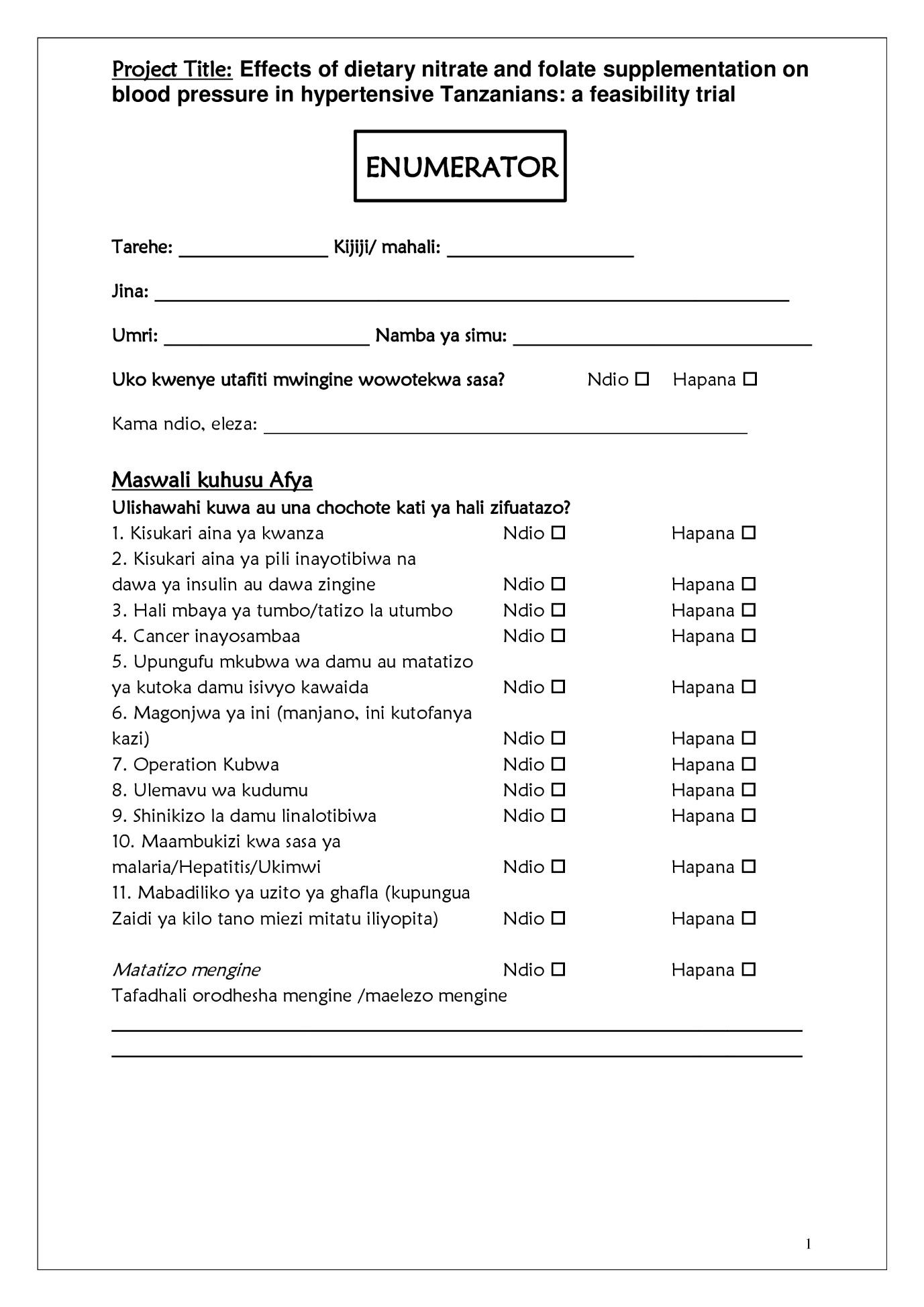

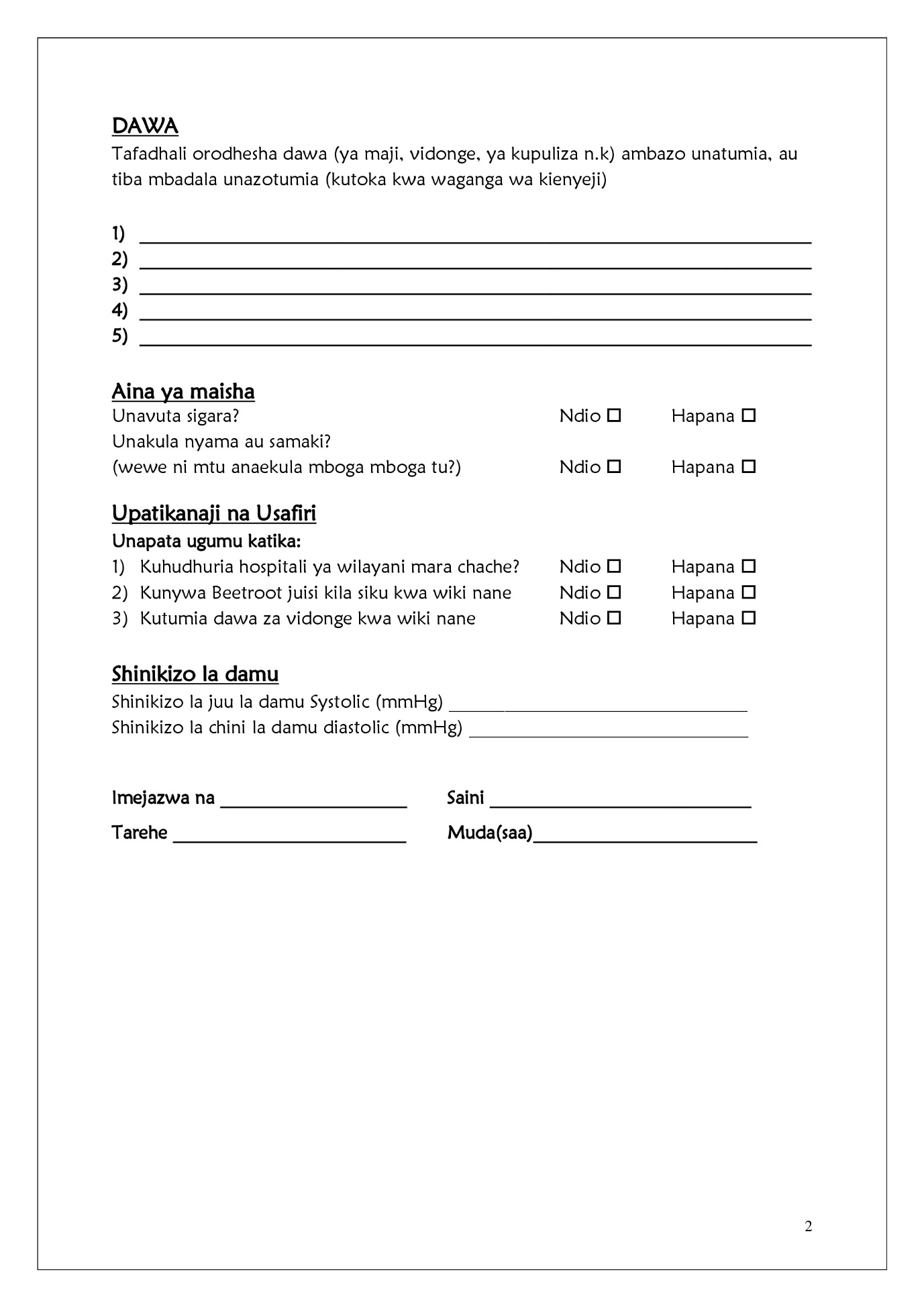


# Appendix 4 – Screening Questionnaire


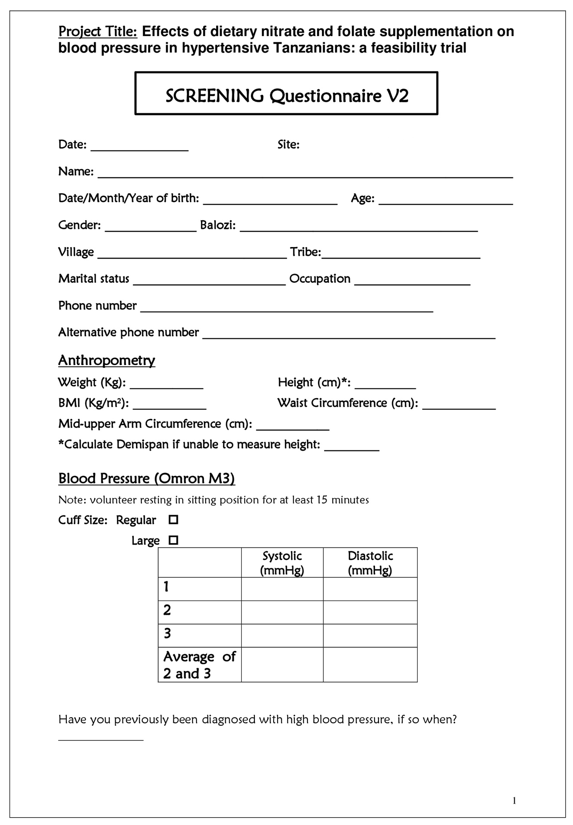


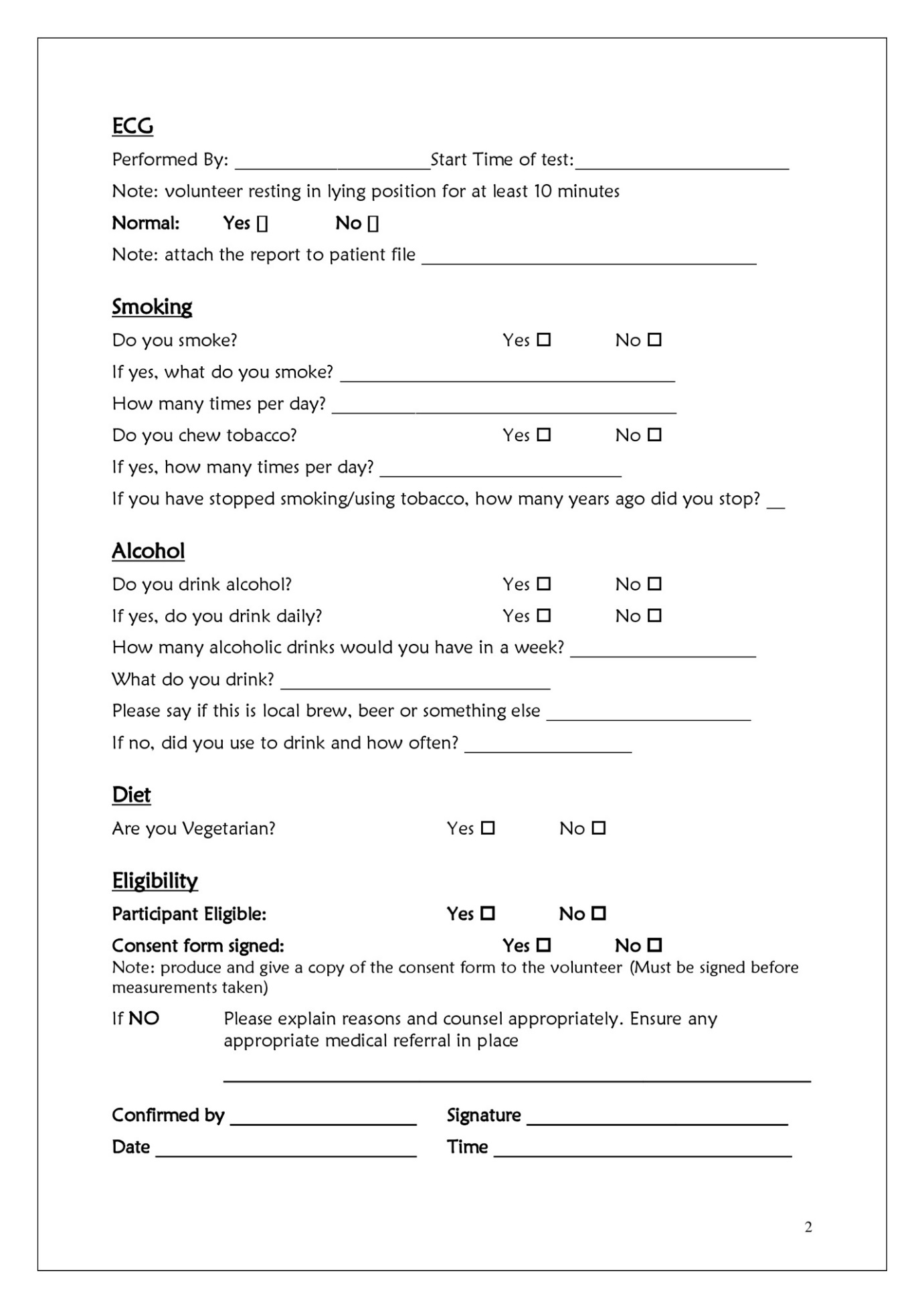


Appendix 5 – Feasibility Questionnaire
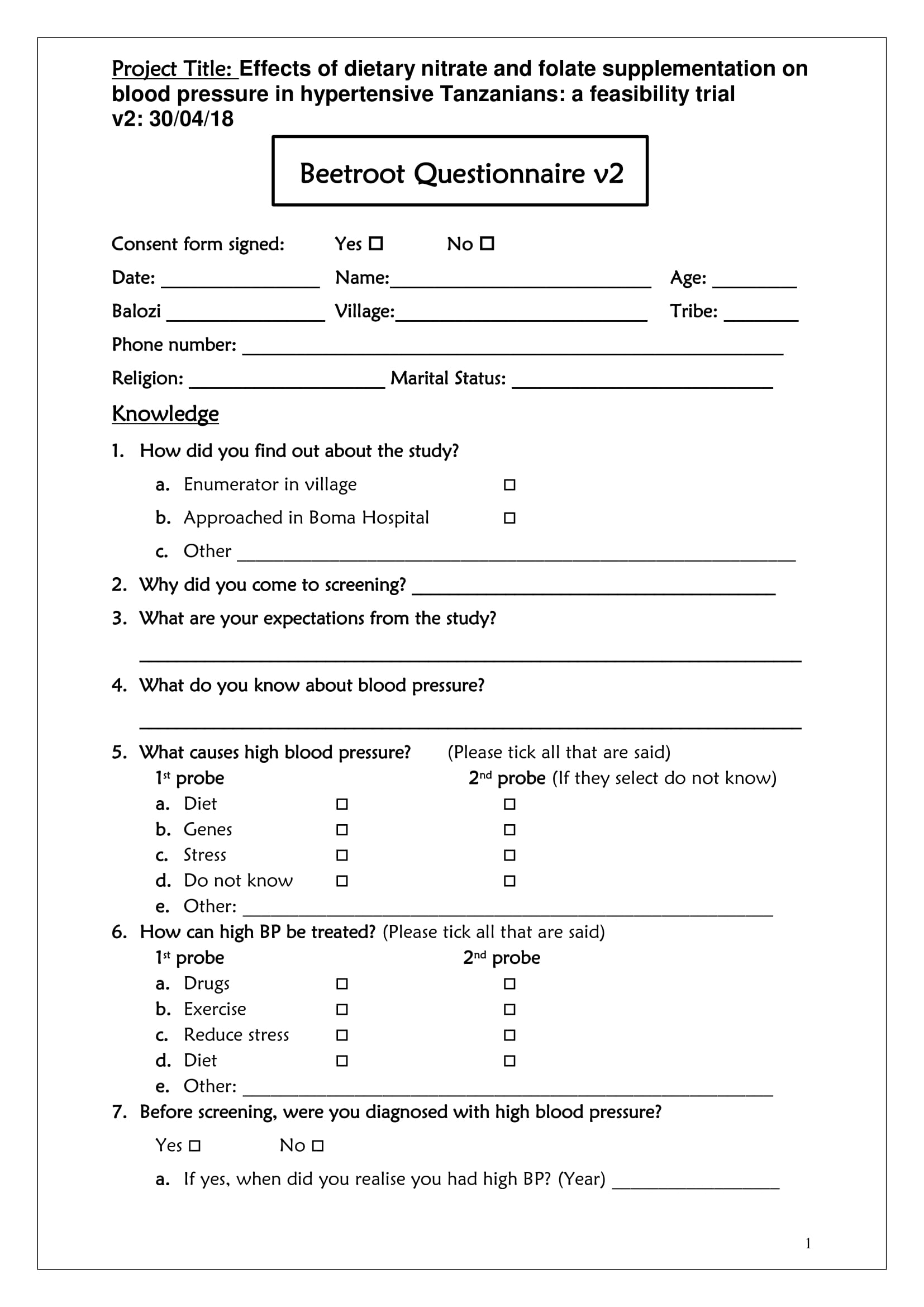

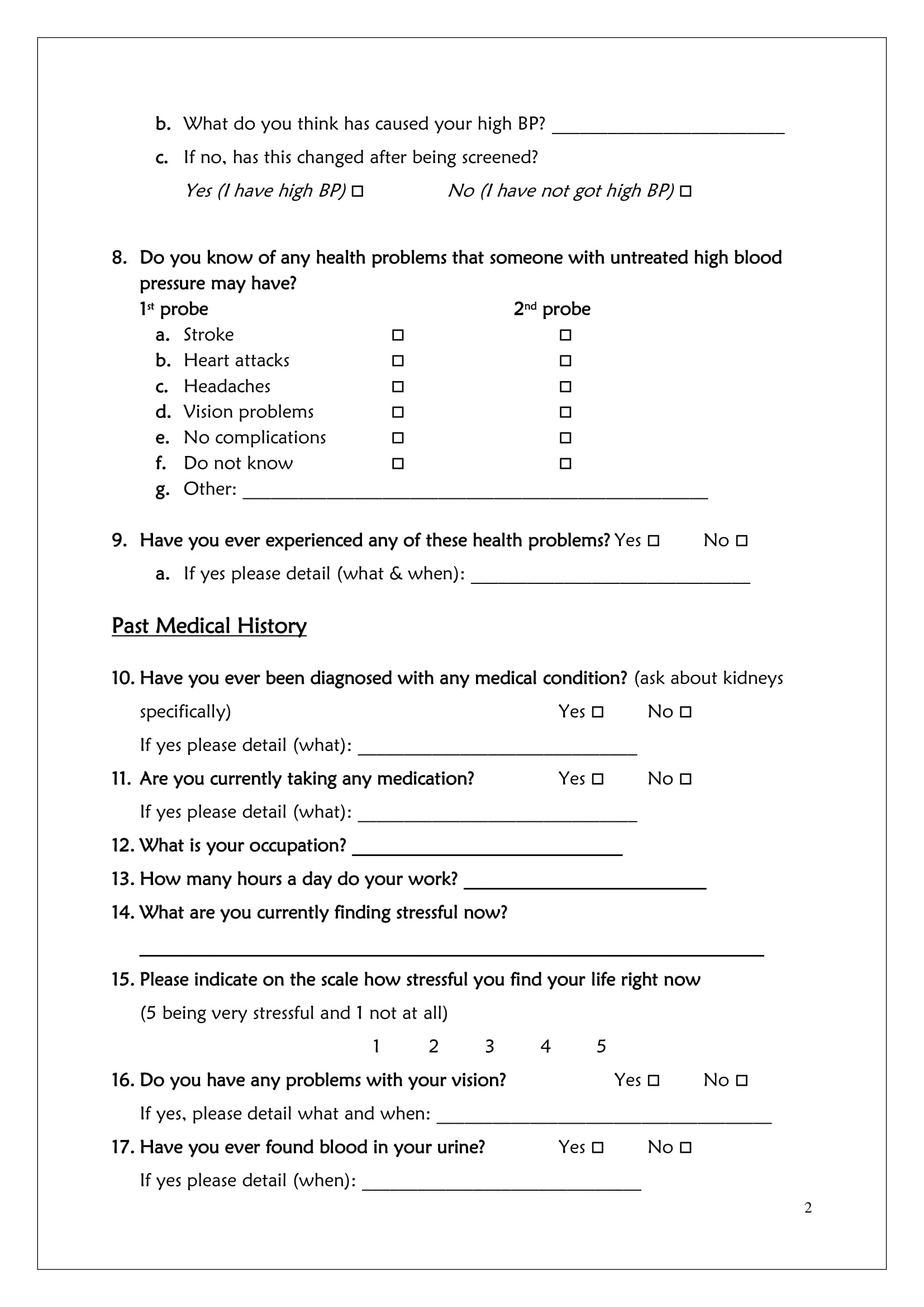

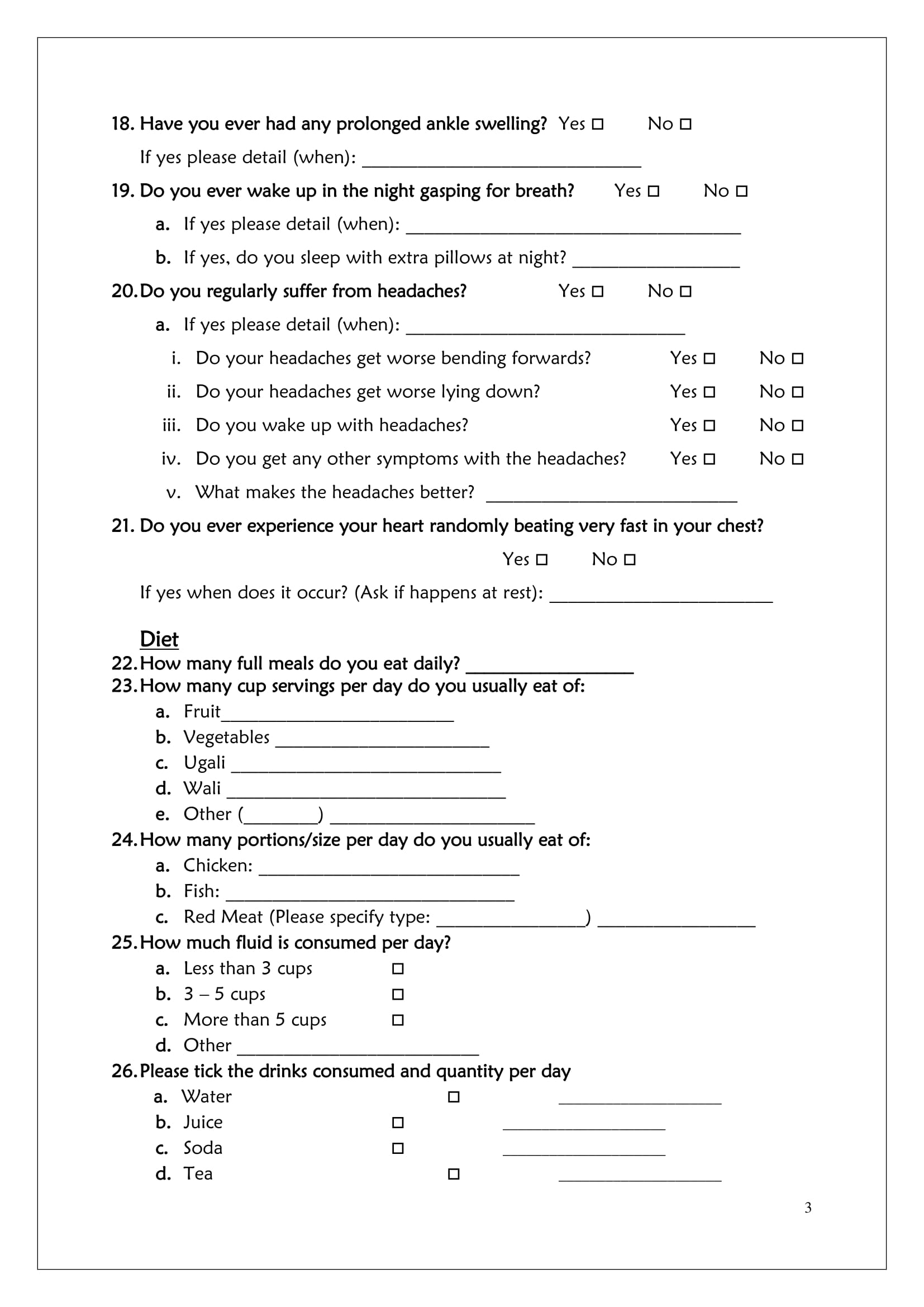

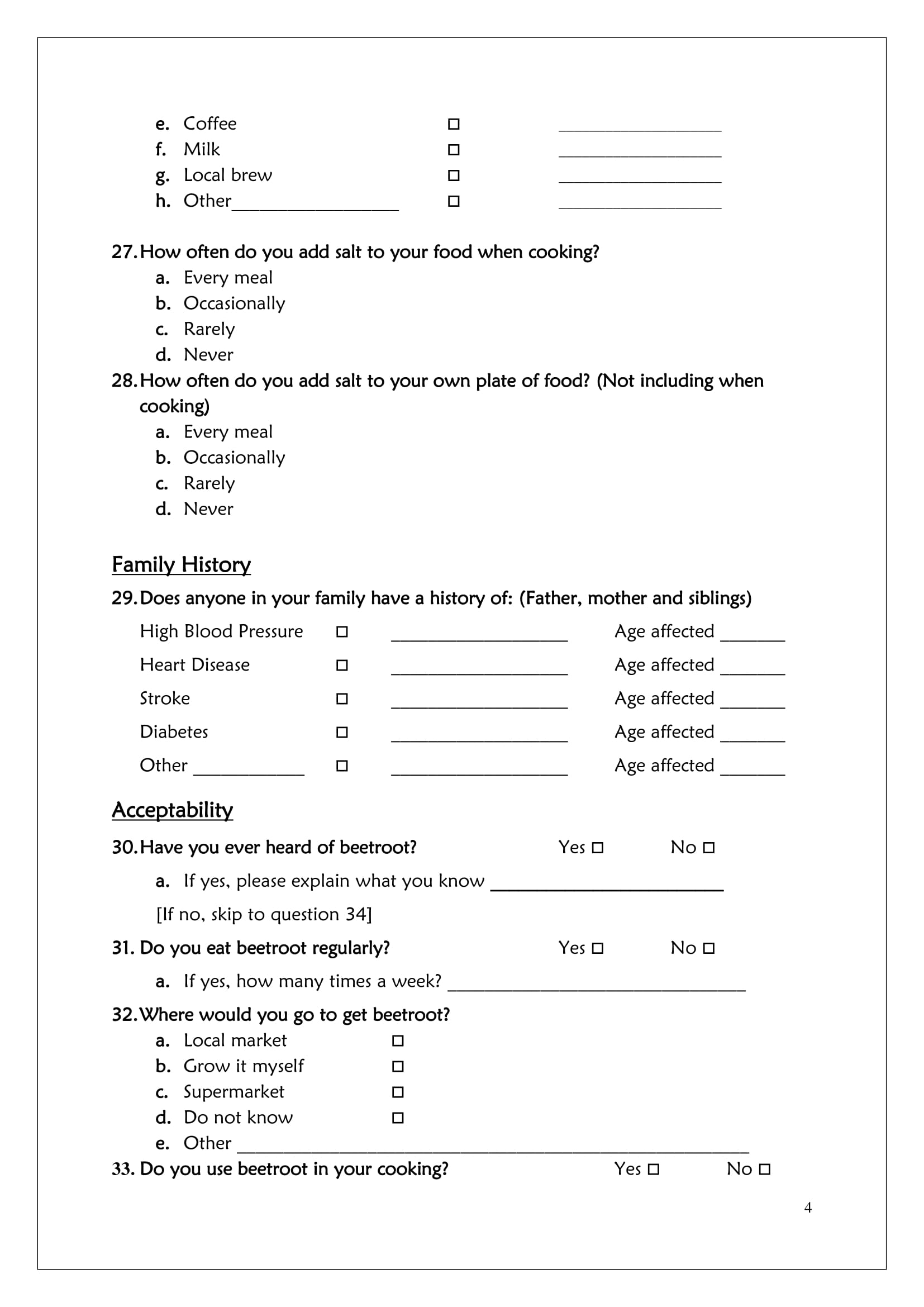

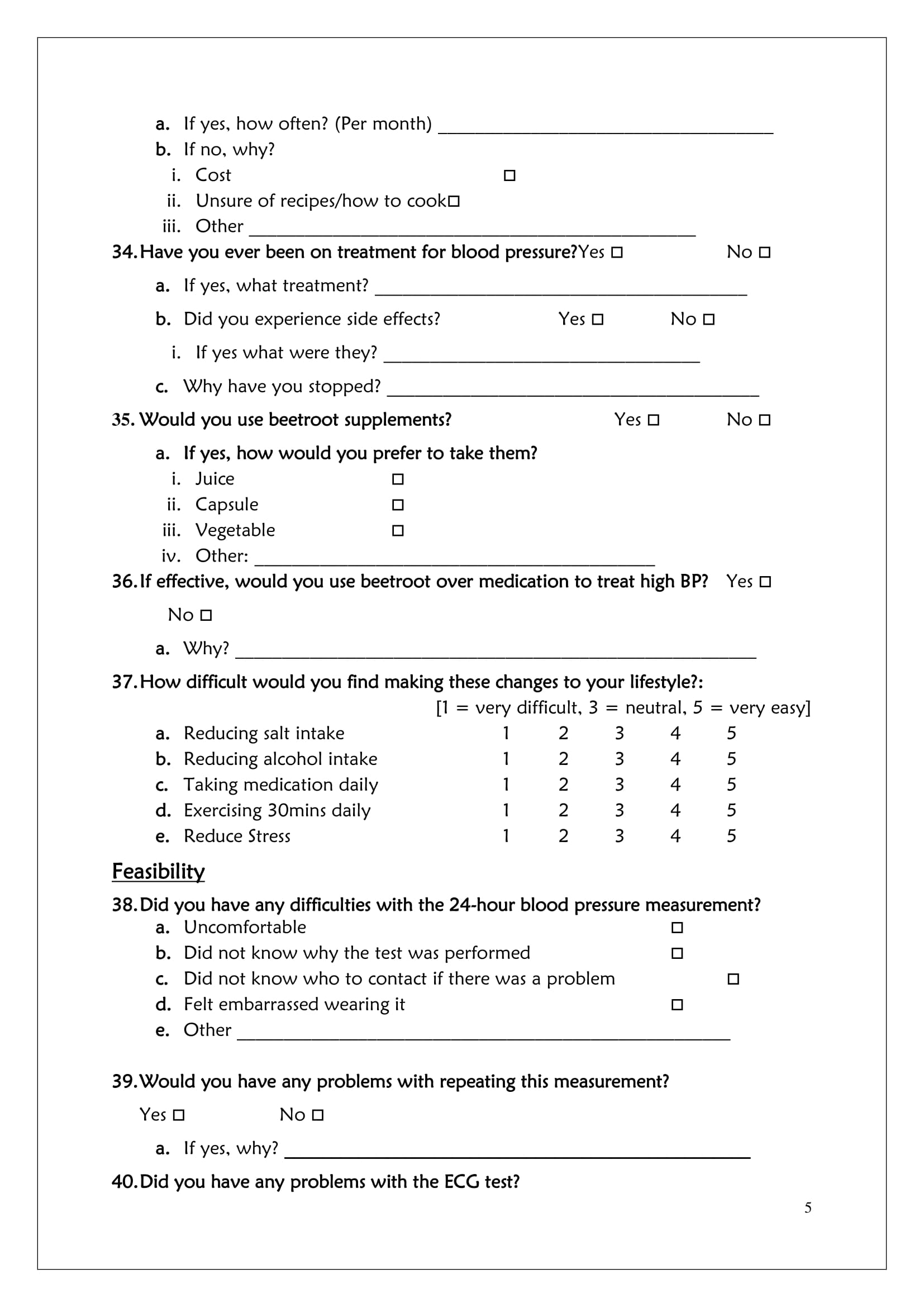


Appendix 6 - International Physical Activity Questionnaire (IPAQ):


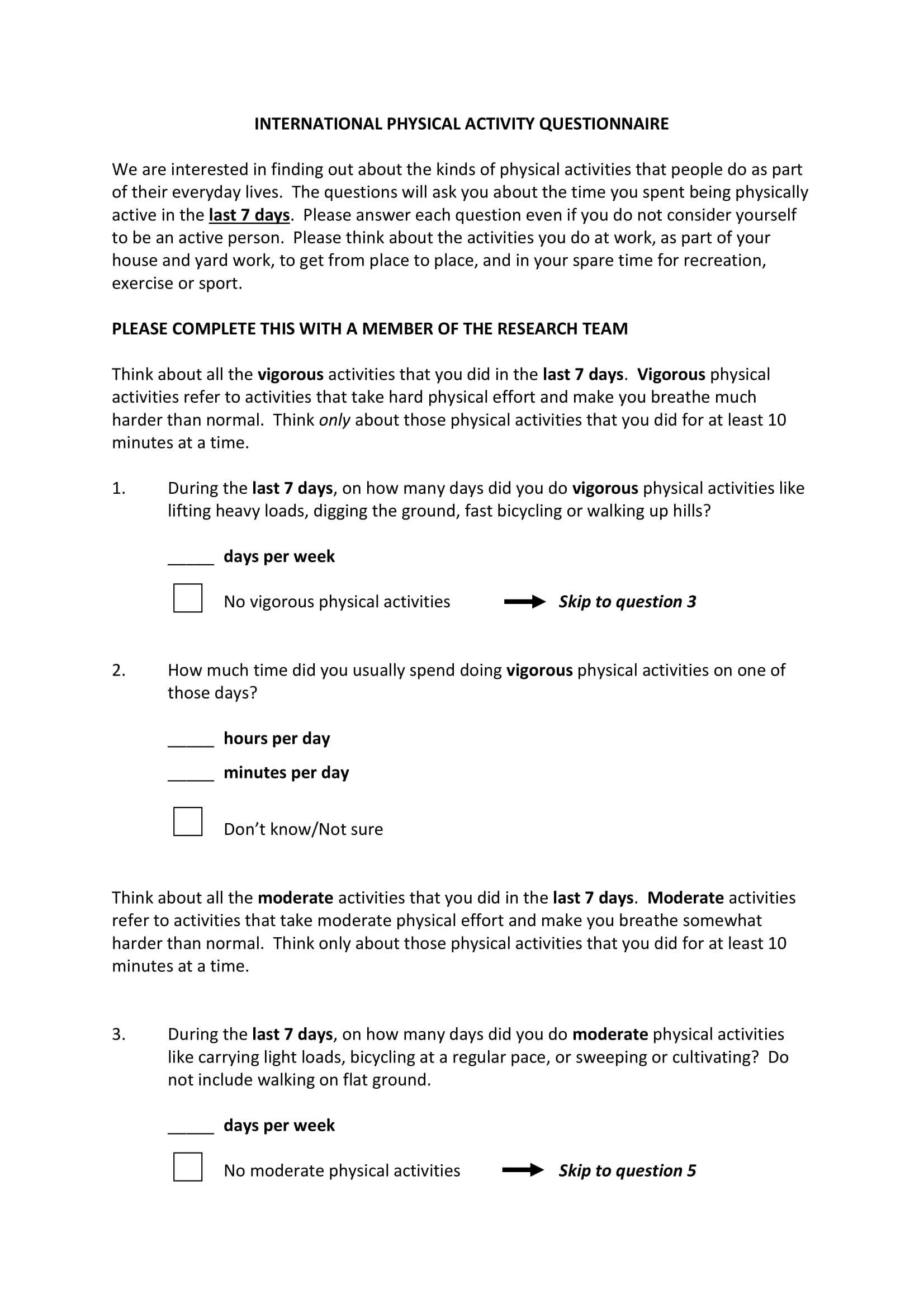


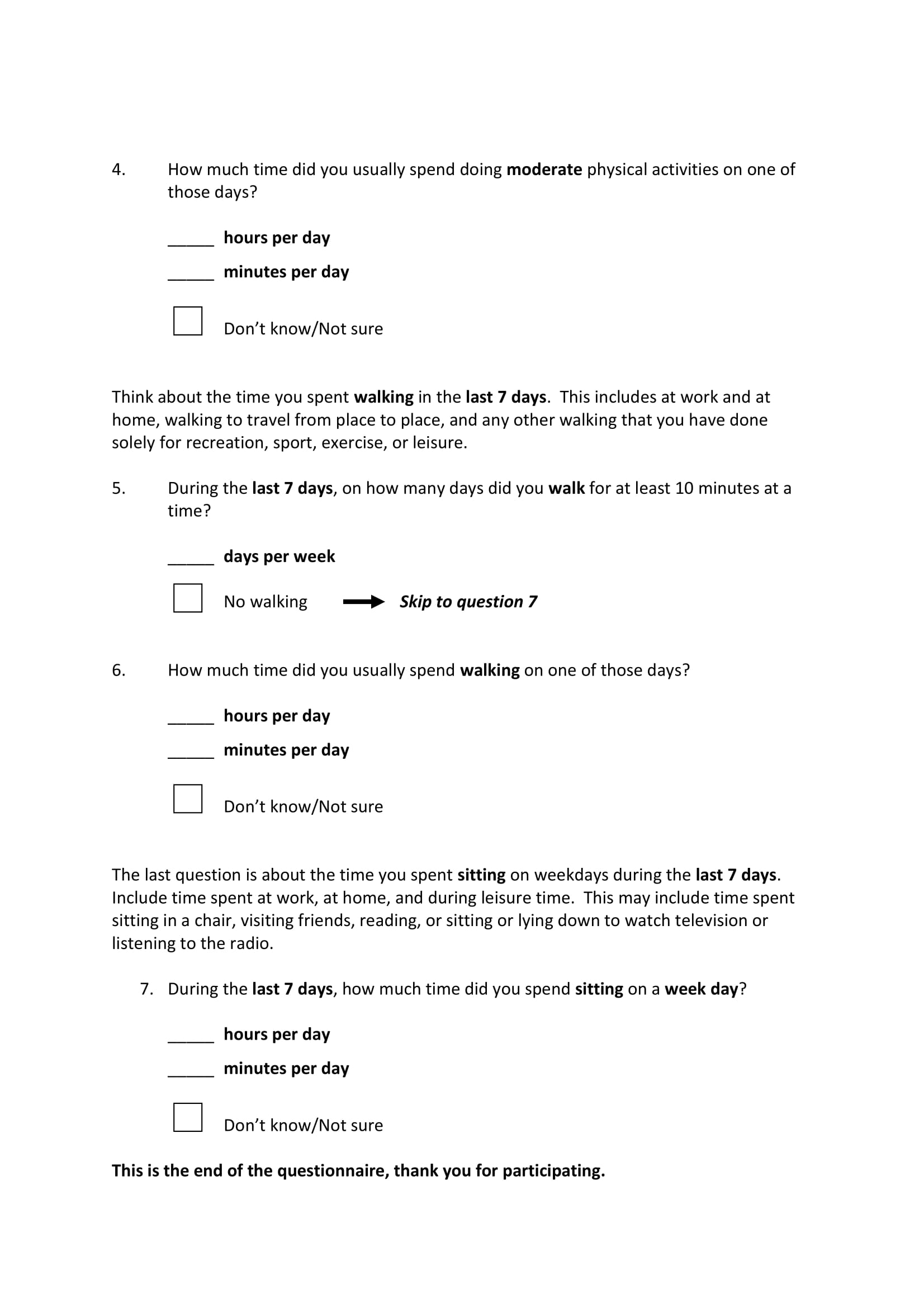


Appendix 7 – Telephone Questionnaire**
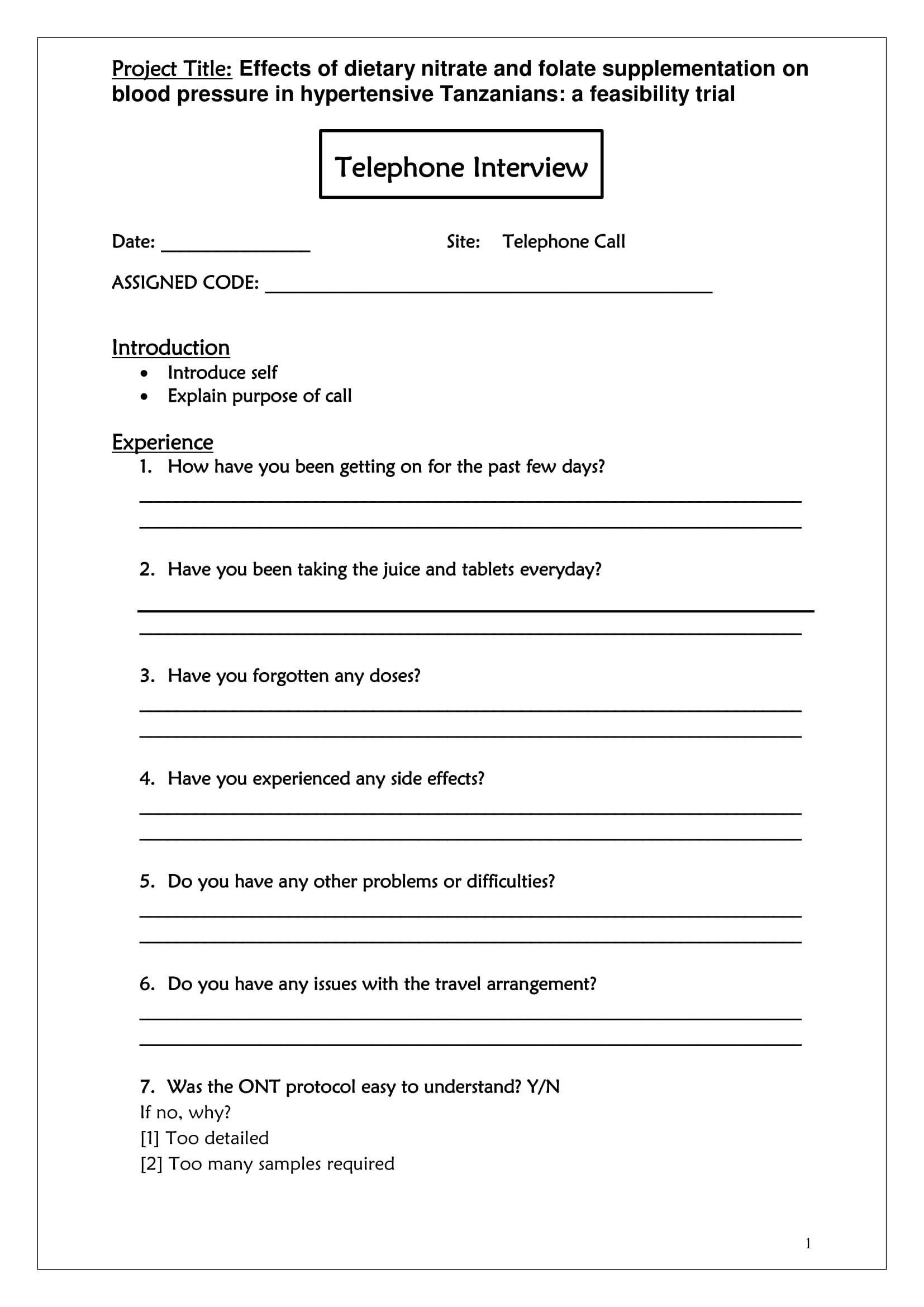

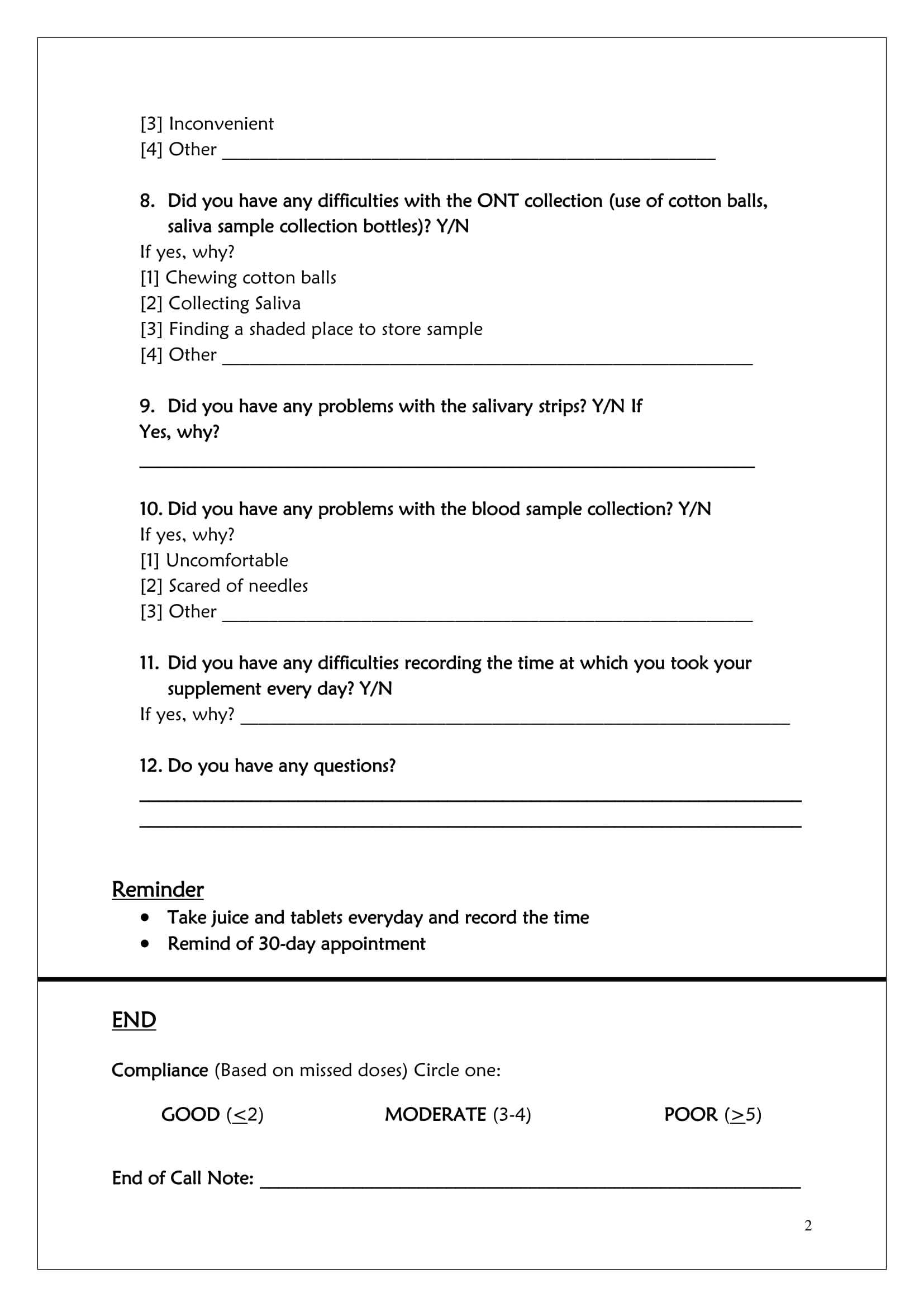
**

Appendix 8 – Short Feedback Questionnaire
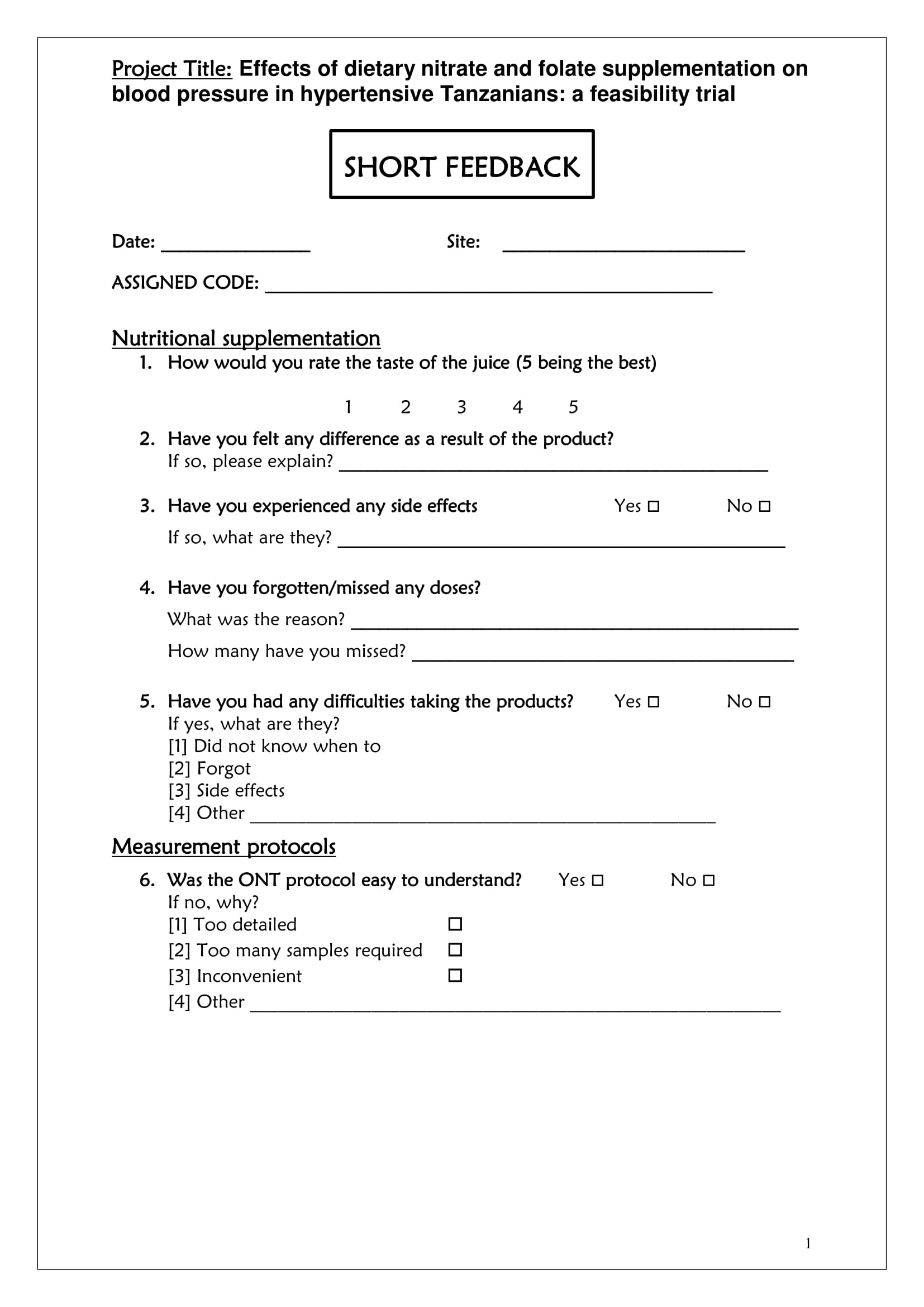


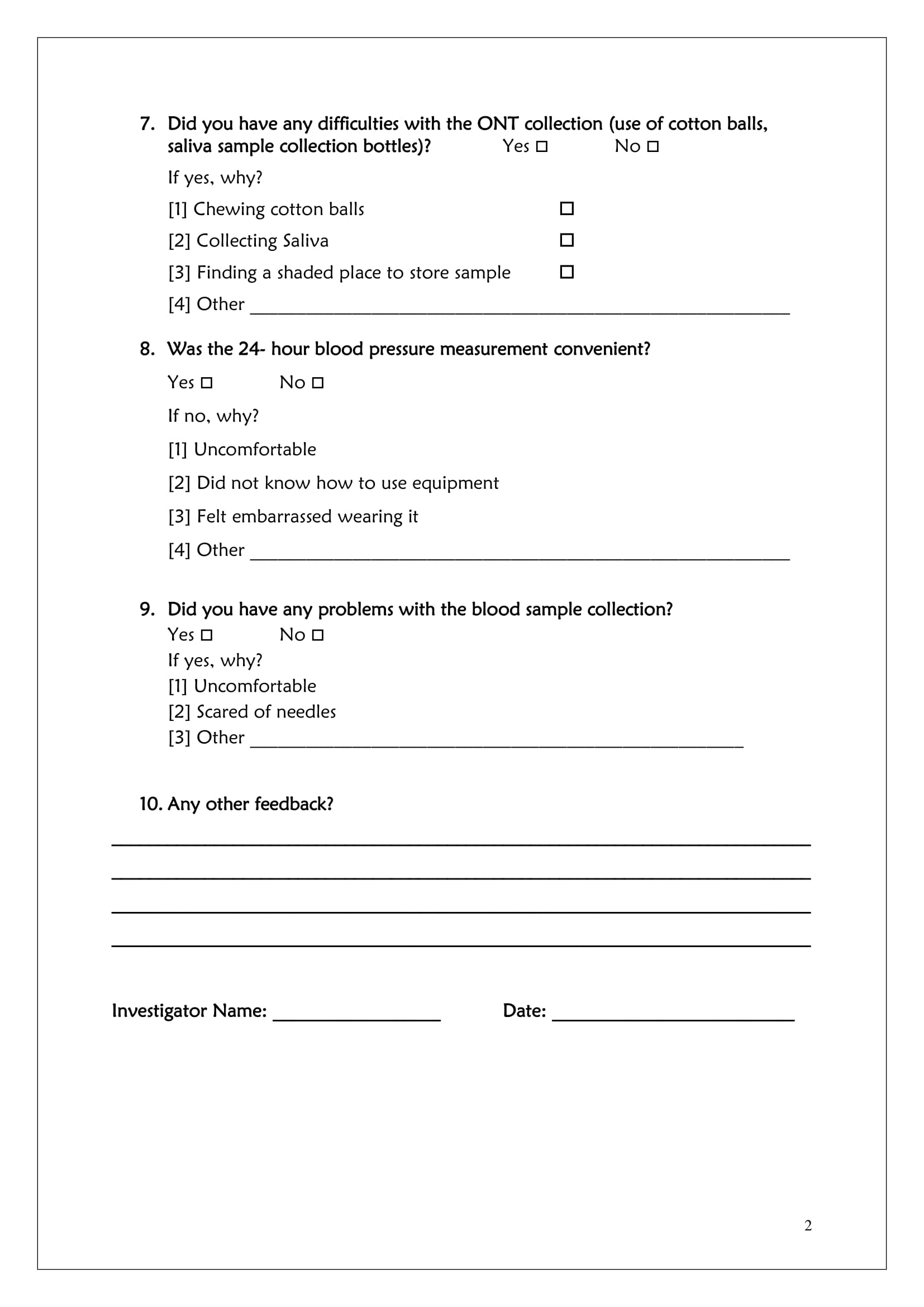


Appendix 9 – Baseline Visit Questionnaire**:**
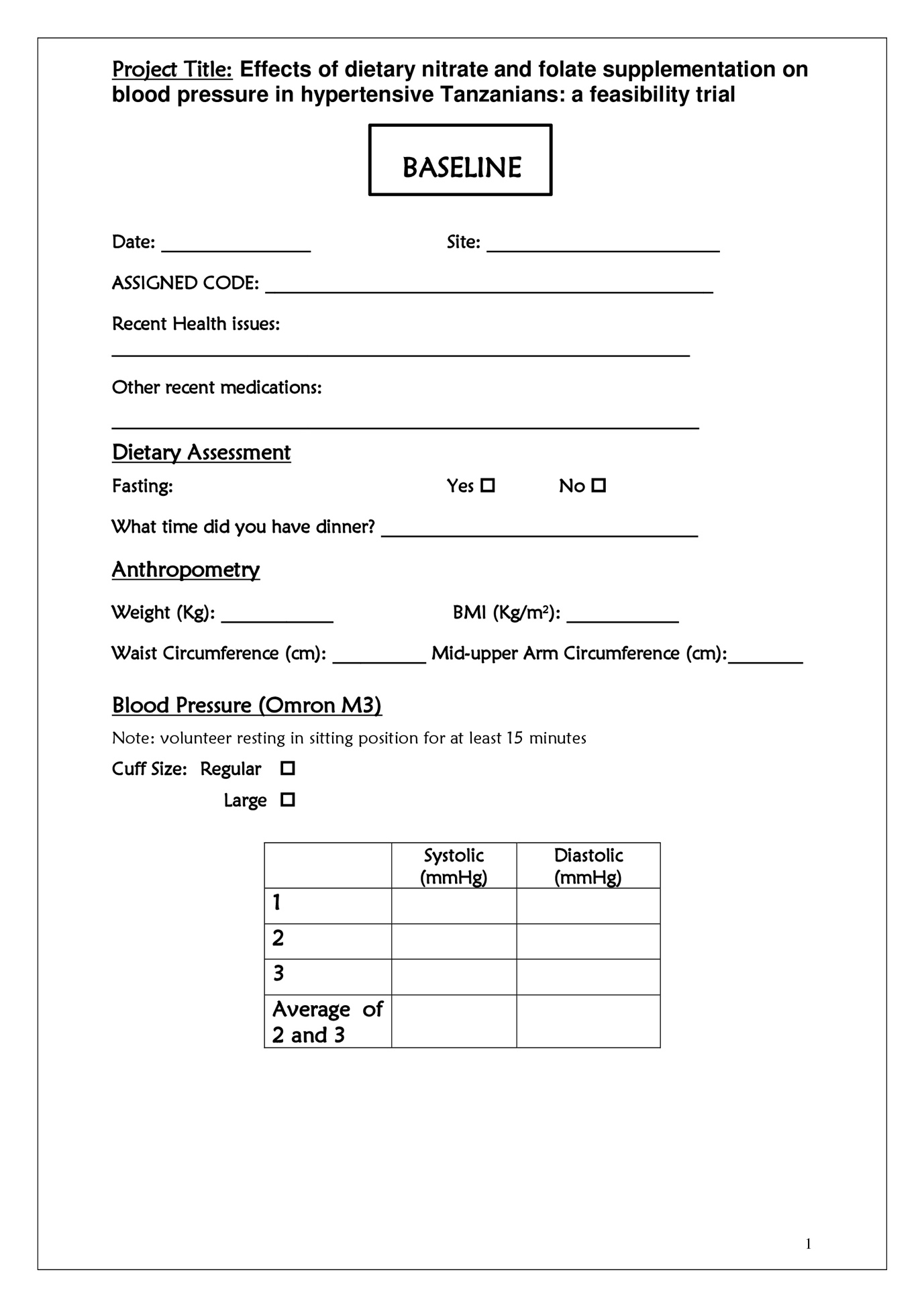


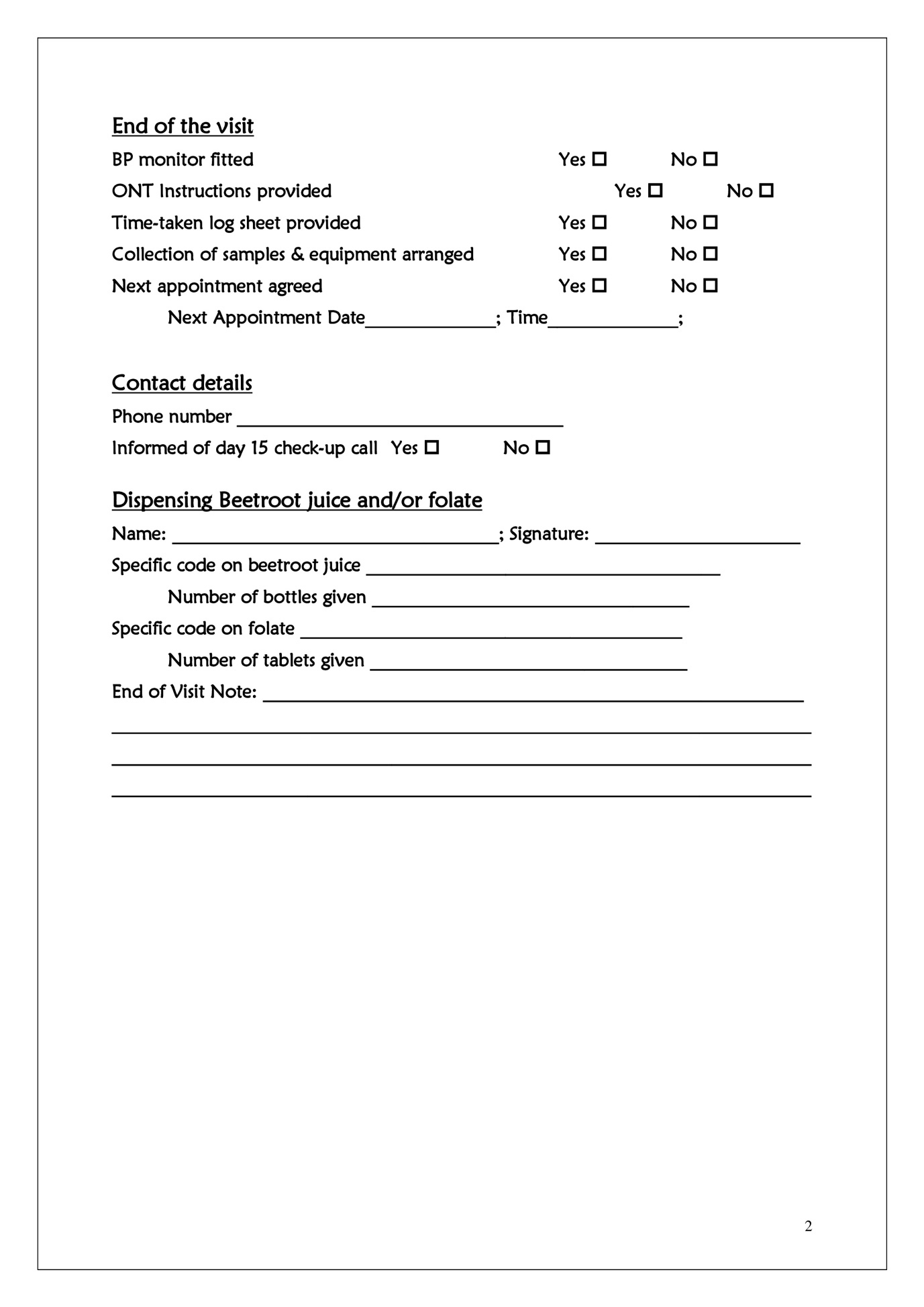


Appendix 10 - 30-day Interim Visit:
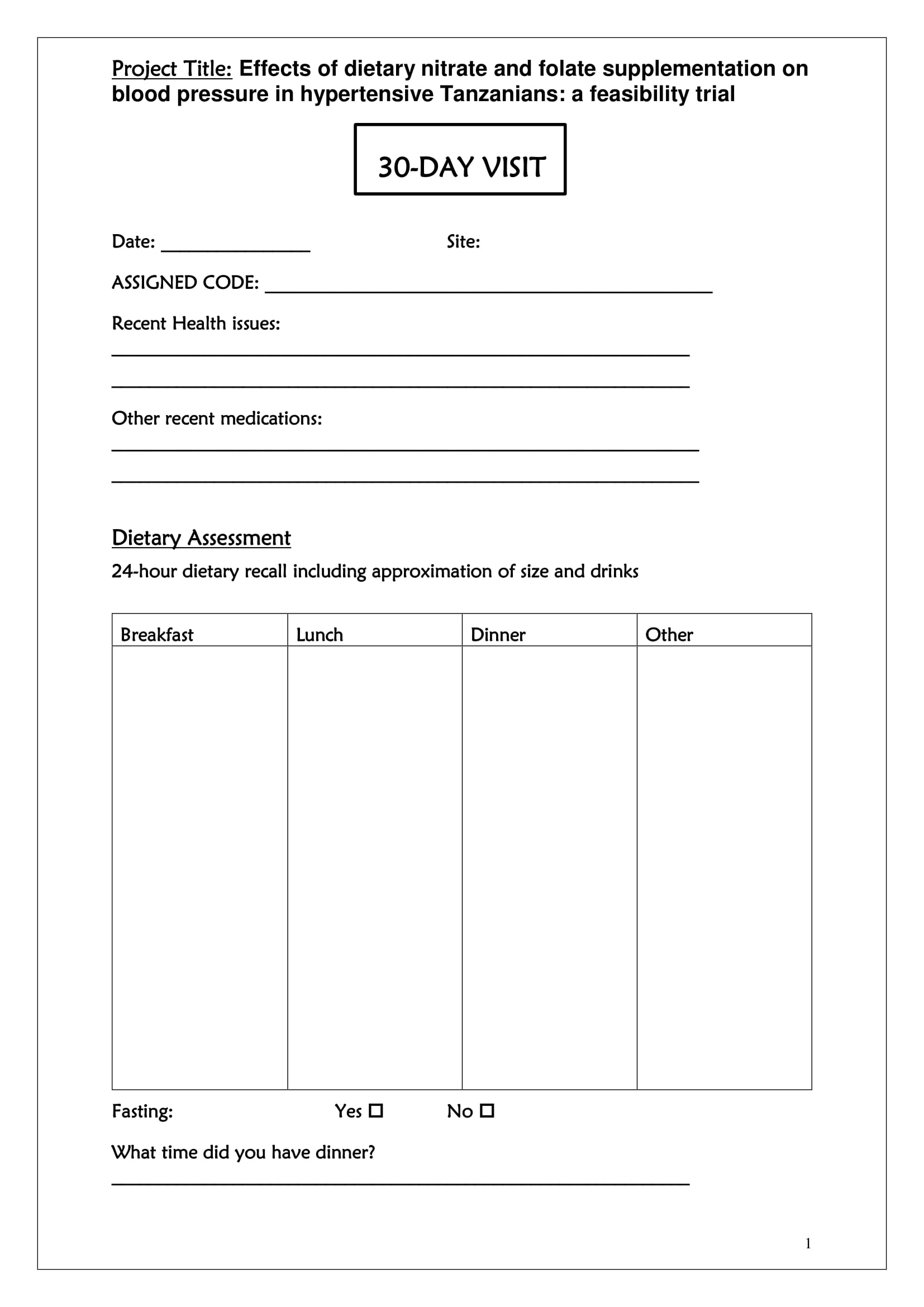


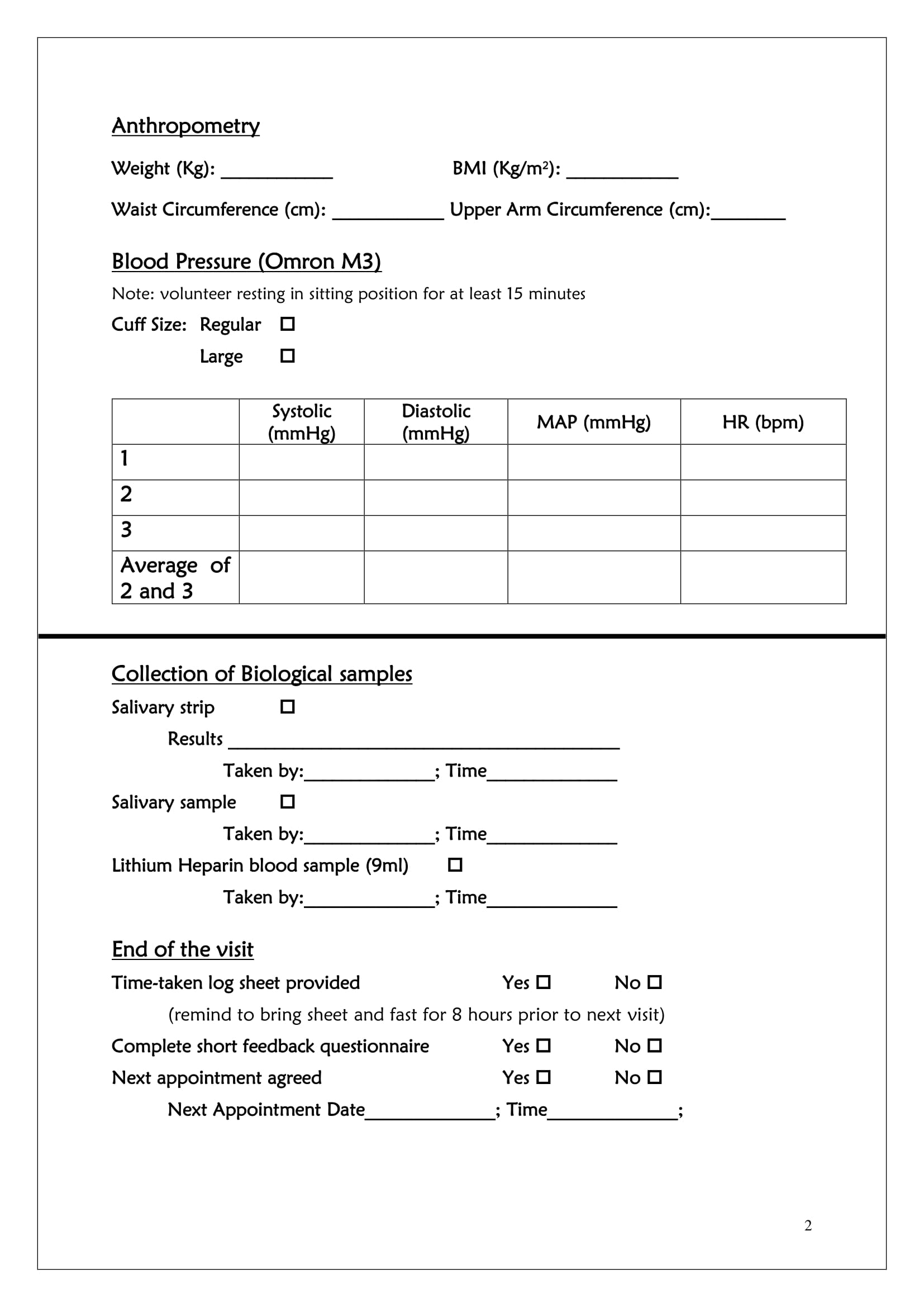

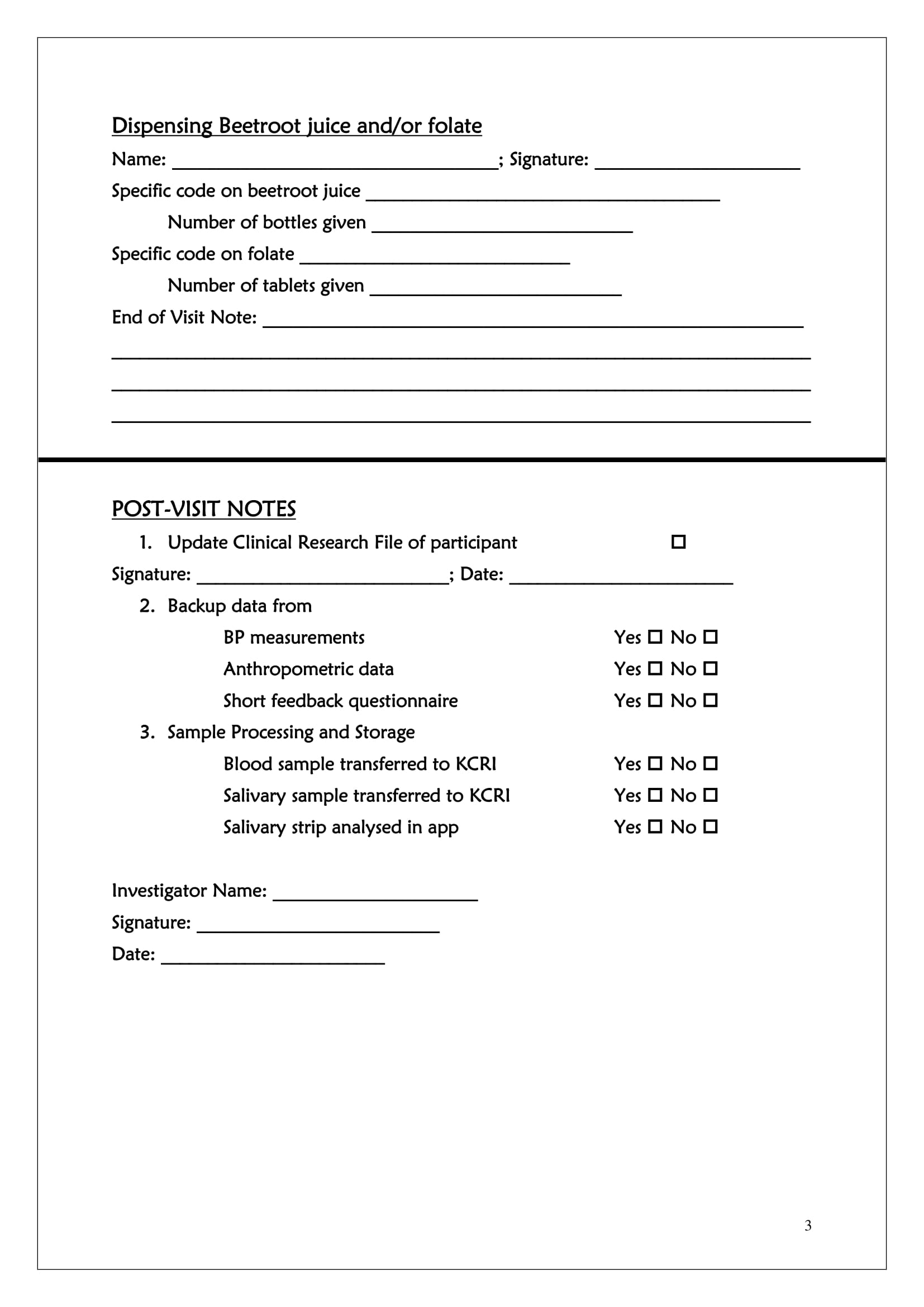


Appendix 11 - Full Feedback Questionnaire
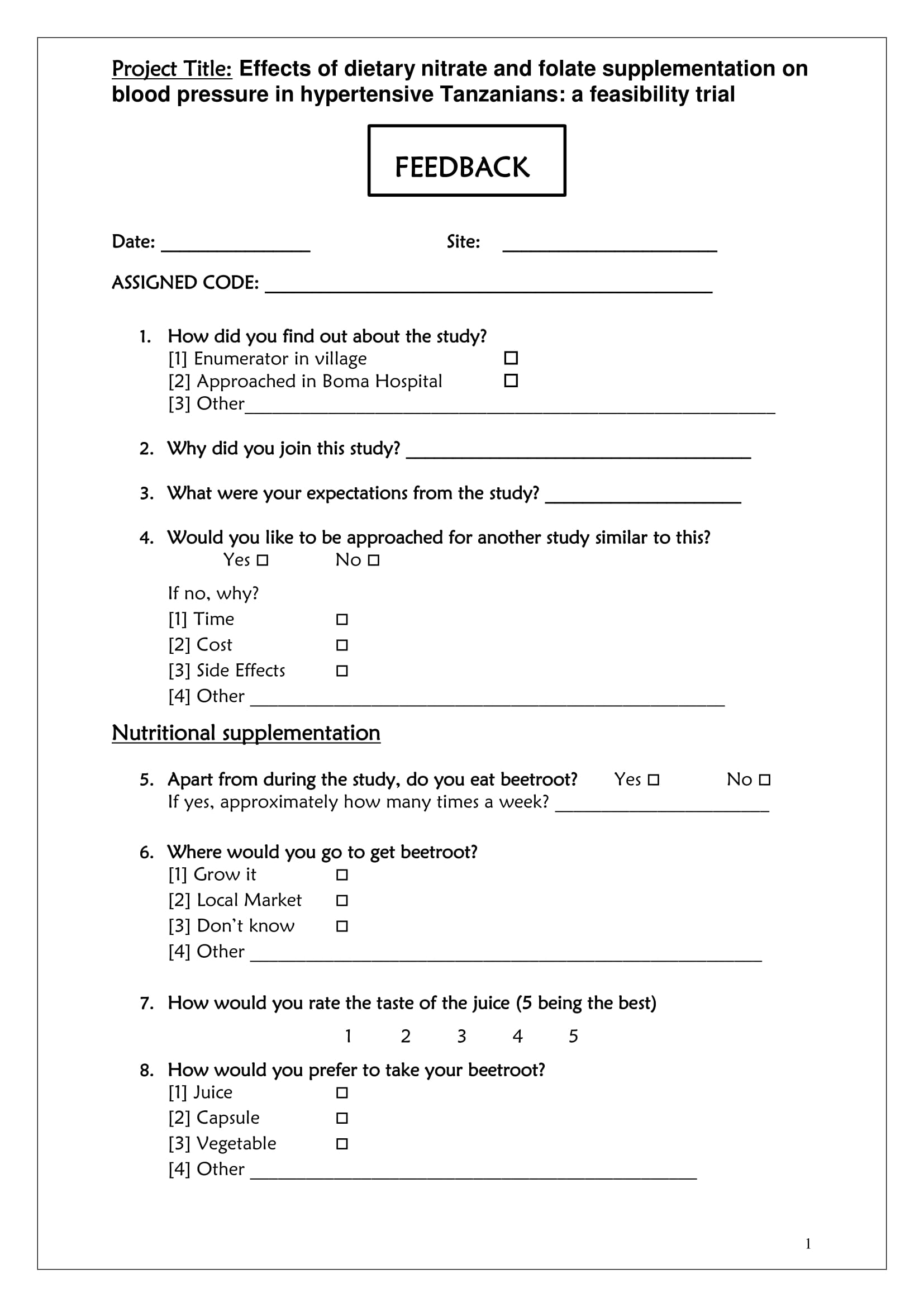


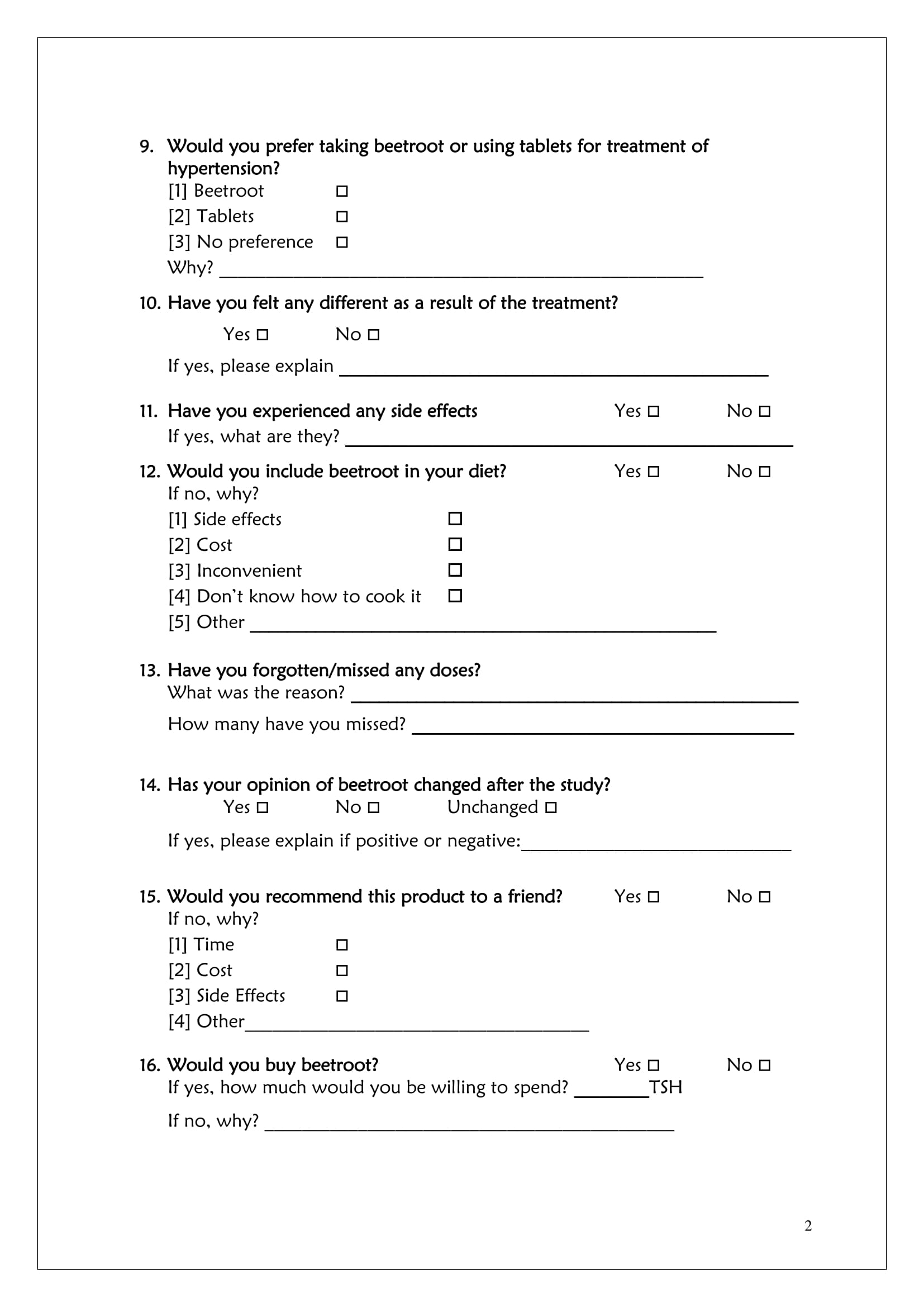

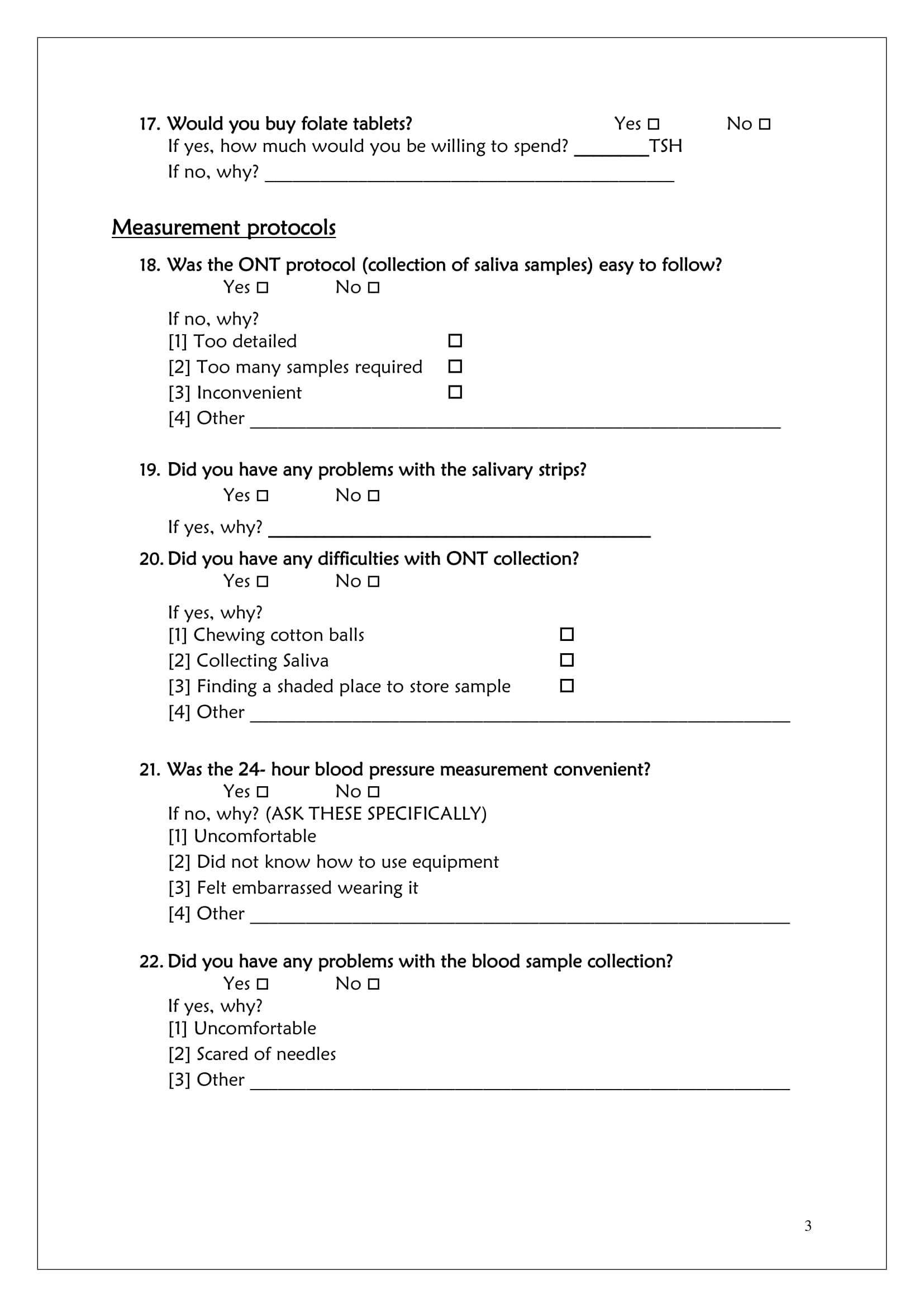


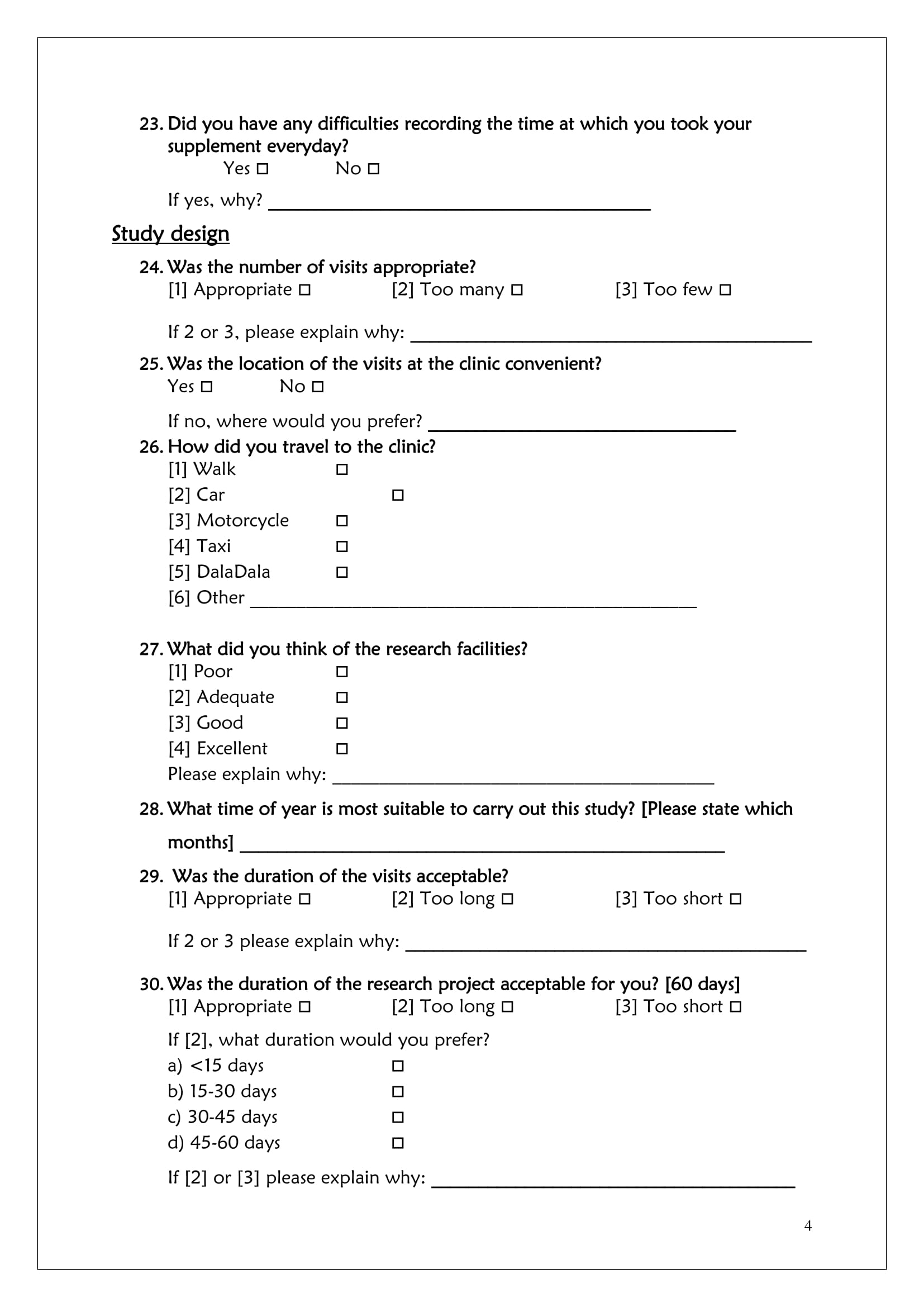


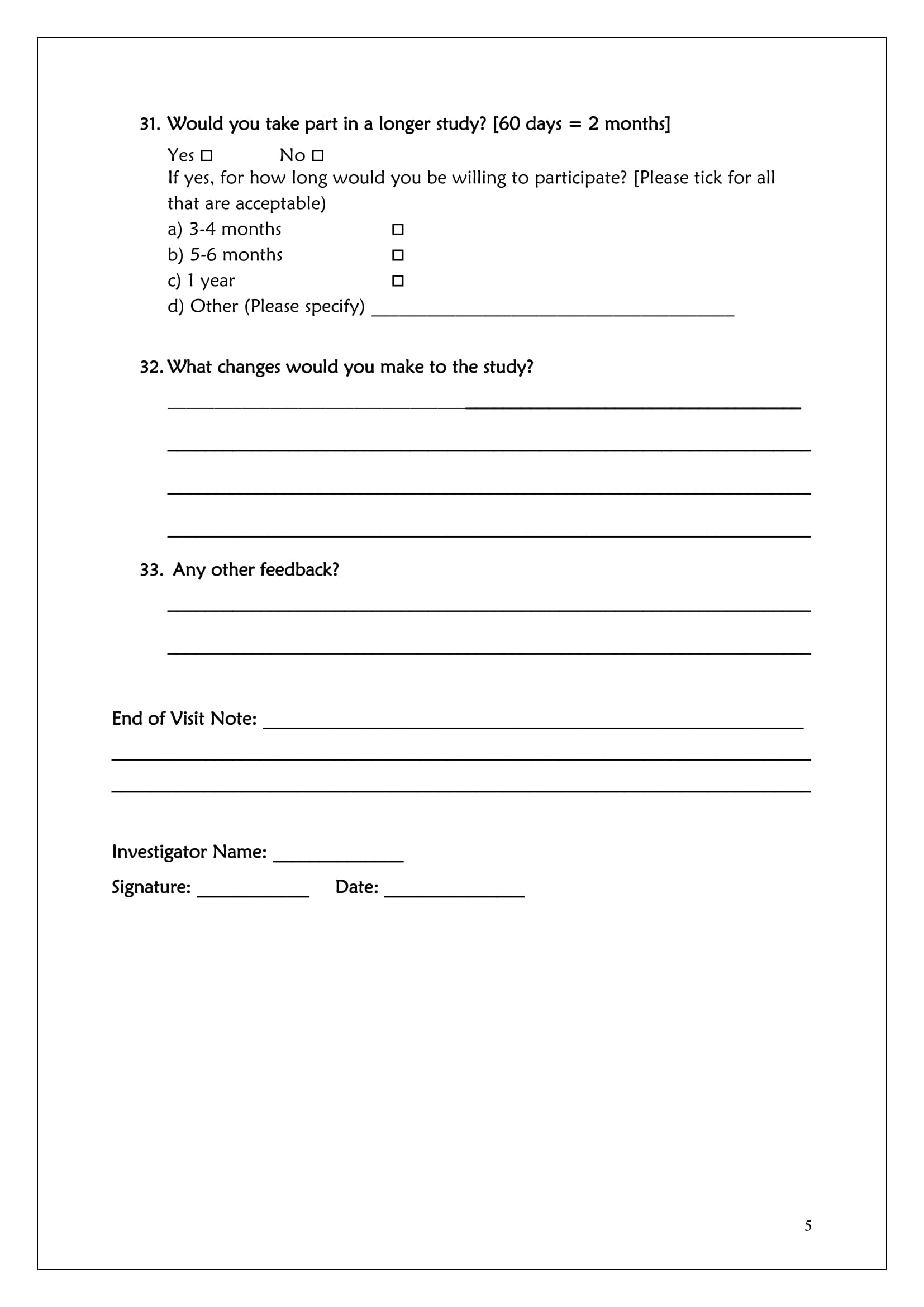

Supplement: Multimedia component 1 [file mmc1.docx]
